# Plasticulture detection at the country scale by combining multispectral and SAR satellite data

**DOI:** 10.1038/s41598-025-93658-2

**Published:** 2025-04-02

**Authors:** Alessandro Fabrizi, Peter Fiener, Thomas Jagdhuber, Kristof Van Oost, Florian Wilken

**Affiliations:** 1 Institute of Geography, University of Augsburg, Augsburg, Germany; 2 Present Address: Microwaves and Radar Institute, German Aerospace Center, Wessling, Germany; 3 Earth and Life Institute, Université Catholique de Louvain, Louvain-La-Neuve, Belgium

**Keywords:** Plastic, Agriculture, Synthetic aperture radar, Optical remote sensing, Sentinel, Google earth engine, Plant sciences, Environmental sciences, Planetary science

## Abstract

The use of plastic films has been growing in agriculture, benefiting consumers and producers. However, concerns have been raised about the environmental impact of plastic film use, with mulching films posing a greater threat than greenhouse films. This calls for large-scale monitoring of different plastic film uses. We used cloud computing, freely available optical and radar satellite images, and machine learning to map plastic-mulched farmland (PMF) and plastic cover above vegetation (PCV) (e.g., greenhouse, tunnel) across Germany. The algorithm detected 103 10^3^ ha of PMF and 37 10^3^ ha of PCV in 2020, while a combination of agricultural statistics and surveys estimated a smaller plasticulture cover of around 100 10^3^ ha in 2019. Based on ground observations, the overall accuracy of the classification is 85.3%. Optical and radar features had similar importance scores, and a distinct backscatter of PCV was related to metal frames underneath the plastic films. Overall, the algorithm achieved great results in the distinction between PCV and PMF. This study maps different plastic film uses at a country scale for the first time and sheds light on the high potential of freely available satellite data for continental monitoring.

## Introduction

**Fig. 1:**
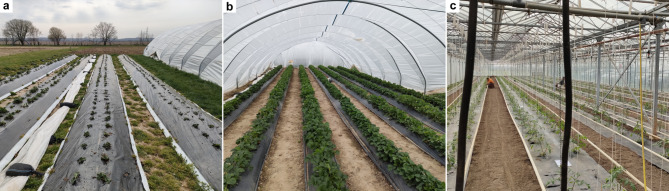
Field pictures of different plasticulture management. From left to right, plastic mulching in open field (**a**), plastic mulching inside a tunnel (**b**), and plastic mulching inside a greenhouse (**c**). Photographs taken by Alessandro Fabrizi.

Plastic plays a key role in modern agriculture by providing many benefits as an inexpensive, lightweight, and resistant material^1^. In European vegetable production, around 80% of plastic consumption is attributed to the use of plastic films for protecting crops^2^ (referred to as plasticulture in this study). While the first crop covers were dated back to Imperial Rome^3^, the use of plastic for protected agriculture started together with the rise of plastic in global markets, in the early 1950s^4^. Nowadays, different types of plastic films may cover either the soil (i.e., mulching) or the vegetation (e.g., tunnels and greenhouses) on around 27 10^6^ ha of agricultural land worldwide^5^, a surface bigger than the United Kingdom. The choice of cover is mainly driven by the crop type and regional climatic conditions. Mulching films are used as soil covers (Fig. 1a), mostly in row crops between seeding and harvest, providing weed and pest control in vegetable crops or ensuring a higher product quality in crops like asparagus. Soil covers also offer reduced evapotranspiration in dry regions or increased soil temperature in cold regions, improving water use efficiency or managing harvest times^6^. Tunnels may offer additional protection for the plants against strong winds or rainfalls (Fig. 1b), reducing the risk of crop loss in soft fruits like strawberry. Greenhouses create a favourable and controlled environment (Fig. 1c), enabling the production of out-of-season fruits and vegetables. Overall, the use of plastic covers delivers benefits that meet the needs of both producers and consumers. Nevertheless, serious concerns have been raised about the end-of-life management of plastic films, especially related to the generation of macro- and micro-plastic residues^7^, which can directly enter the soil matrix or be transported to other environmental matrices through water or wind erosion^8,9^. Heavily plastic-polluted soils in experimental setups often exhibited degradation of physiochemical properties, biome, and plant health, whereas the threshold concentrations at which the effects occur remain unclear^10^. Likewise, the contribution of plastic films to plastic concentrations in soil remains unclear but film thickness has been identified as one of the major drivers of the amount of residues in soil^7^. End-of-life management influences also disposal and recycling rates, which vary depending on the type of film and are usually lower when the films are used in direct contact with the soil^11,12^. Altogether, management practices, crop type, and plastic film manufacture are some of the variables that can affect the plastic footprint of covered crops. Despite a frequently combined use (e.g., mulching films under greenhouses or tunnels) and the increasing use of biodegradable plastics, mulching films are estimated to have a higher environmental risk compared to greenhouse films^13^.

In the context of assessing the plastic footprint of agriculture, further studies are needed to quantify and monitor the extent of different plasticultural management practices. The available data are mostly related to the amount of plastic films produced and, at best, sold to individual countries, thus hindering the retrieval of a spatial distribution. A few estimates of the area covered by plastic films were derived either from these data or other sources such as agricultural surveys. Yet these data sources are scarce and scattered in time and space, and still limited to a regional or national resolution. A tool for high-resolution detection and monitoring of the area covered by different plastic covers at a large scale (e.g., regions up to continents) is still lacking.

Satellite remote sensing has been qualified as an effective tool for plasticulture mapping and a wide range of different methodologies with overall accuracy ranging between 85 and 98% has been developed^14^. However, the great majority of the studies focused on plasticulture hotspots associated with a specific plastic cover type, like greenhouses in Almeria, Spain^15,16^ or plastic-mulched cotton fields in northern Xinjiang, China^17,18^. Some authors reported great accuracies with techniques like object-based image analyses^16,19^, which require computational resources that cannot easily be coupled with large-scale analysis. Some others developed new spectral indexes for pixel-based approaches^17,20^, but classification techniques based on these spectral indexes often depend on thresholds that vary based on the site or the applied remote sensing dataset^21^. Indeed, besides some absorption features in the infrared region that belong to sensors on board of commercial satellites^22,23^, plastic film spectral signature might differ based on manufacturing parameters (e.g., thickness, additives, and colour)^24^ or extrinsic factors like underlying soil and vegetation growth^25^. Larger study regions increase the heterogeneity of all these variables and the possibility of finding features with spectral similarity like impervious surfaces or cultivations under glass.

The interest in the upscaling of plasticulture mapping has been mainly directed towards plastic greenhouses^26–28^, while large-scale detection of plastic-mulched farmland (PMF) remains mainly unexplored and limited to regions where mulching films are associated with a specific crop or plastic-type, like transparent films used for cotton fields^29^. Moreover, PMF detection has been restricted to seasonal and site-specific temporal windows so far, given their temporary use as soil covers coupled with vegetation growing above the films. Synthetic Aperture Radar (SAR) cloud penetration potentially increases these temporal windows and makes them extremely attractive to PMF mapping. Despite their low individual performances in detecting PMF, their use in a multi-sensor approach increases the performance of optical data^30^. Nonetheless, SAR data have never been used for the detection of plastic covers above vegetation (PCV), such as greenhouses or tunnels^21^.

In this research study, we used Google Earth Engine (GEE)^31^ to combine yearly time series of densely recorded Sentinel-1 (S1) and Sentinel-2 (S2) observations and classify German agricultural land based on three different land use classes: plastic-free farmland (PFF), PMF, and PCV. In this way, we aim at developing for the first time a methodology to enable large-scale detection and monitoring of PMF and PCV, using open satellite data.

## Methods

### Study area

**Fig. 2:**
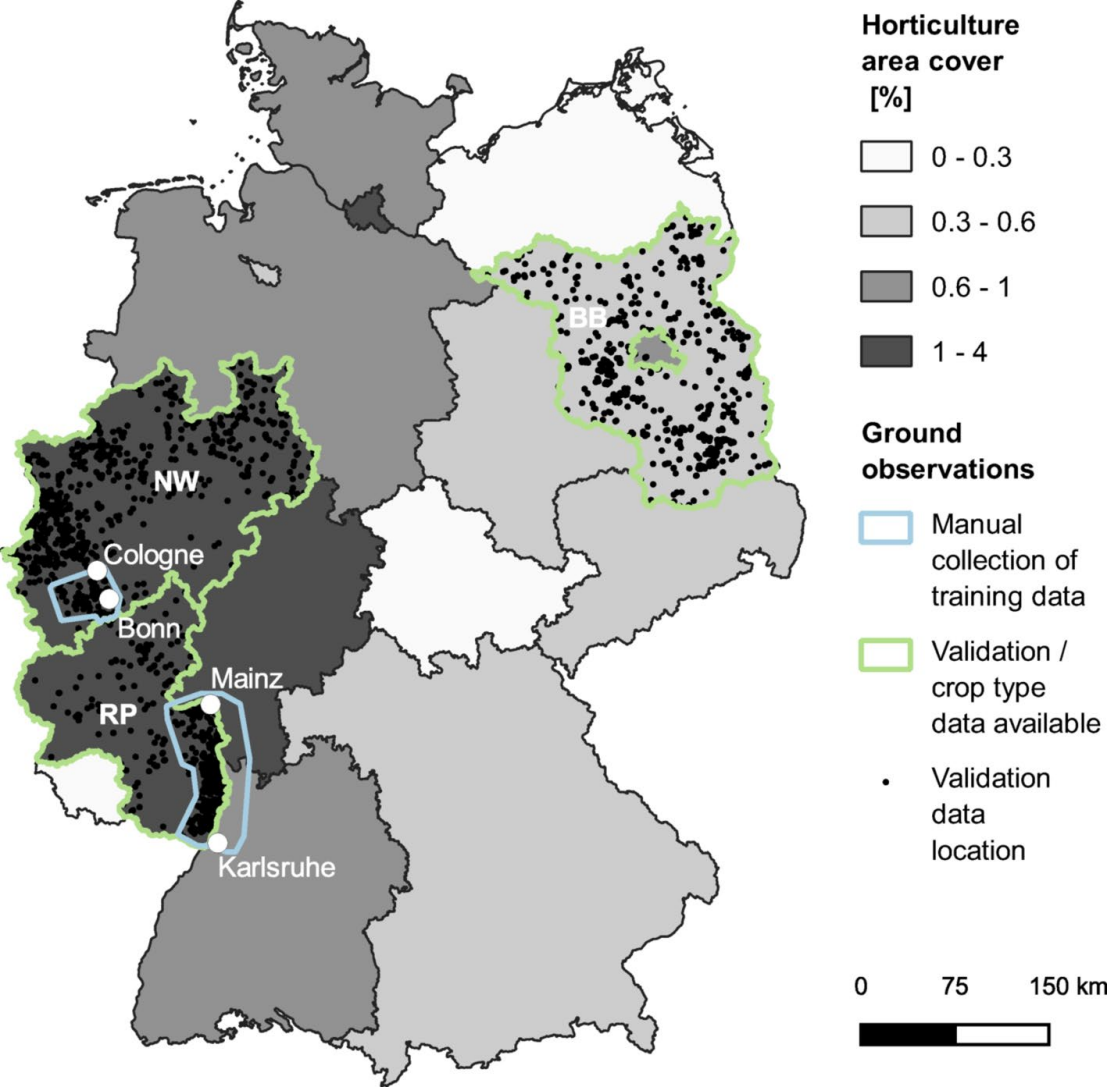
Study area (Germany) divided by Federal States. On a grey scale, the horticulture area relative to the whole of the agricultural land. In blue, the two regions where the training data were manually collected. In green, the three Federal States where the validation data were collected and where GSA data were available. BB: Brandenburg; NW: North Rhine-Westphalia; RP: Rhineland-Palatinate. Map created using QGIS 3.12^64^.

In the EU, Germany has the third largest national territory covered by agricultural land use^32^ and the third highest estimated annual sales of agricultural films^2^. The use of plastic films for crop covers in Germany is primarily intended for the horticultural sector (vegetables, strawberries, and other garden plants), which in 2020 occupied 140.4 10^3^ ha^33^. The relative area occupied by horticulture in every state increases along a West–East gradient (Fig. 2), with an average value of 0.8% of the total agricultural land. Moreover, the mean agricultural holding size (following the definition in Eurostat^34^) for horticulture in Germany is 9.2 ha, while the mean for the agricultural land is 63.1 ha. Therefore, despite the relevant quantities of agricultural films used in Germany at the EU level, plasticulture is distributed on a relatively small area and generally managed on smaller scales, compared to the entire agricultural land.

More in-depth evaluations of the results were possible in the Federal States of Brandenburg, North Rhine-Westphalia, and Rhineland-Palatinate (Fig. 2), due to the availability of crop type information at the parcel level. North Rhine Westphalia has the biggest horticultural area in Germany (31.5 10^3^ ha). At the same time, it is the Federal State where horticulture has the highest relative area compared to its overall agricultural land (1.9%). Rhineland-Palatinate stands right behind, with 1.7% (13.4 10^3^ ha) of the agricultural land occupied by horticulture, while Brandenburg has just 0.5% occupied by horticulture, corresponding to 6.9 10^3^ ha. In contrast, Brandenburg exhibits the highest mean agricultural holding size for horticulture among these states (18.6 ha), followed by Rhineland-Palatinate (17.9 ha) and North Rhine-Westphalia (11.6 ha), having implications on the number of agricultural holdings and potential differences in terms of heterogeneities in field management practices and crops cultivated. Overall, these three regions represent together around 21% of German agricultural land and 37% of the land cultivated with vegetables, strawberries, and other garden plants^33^.

### Workflow overview

**Fig. 3:**
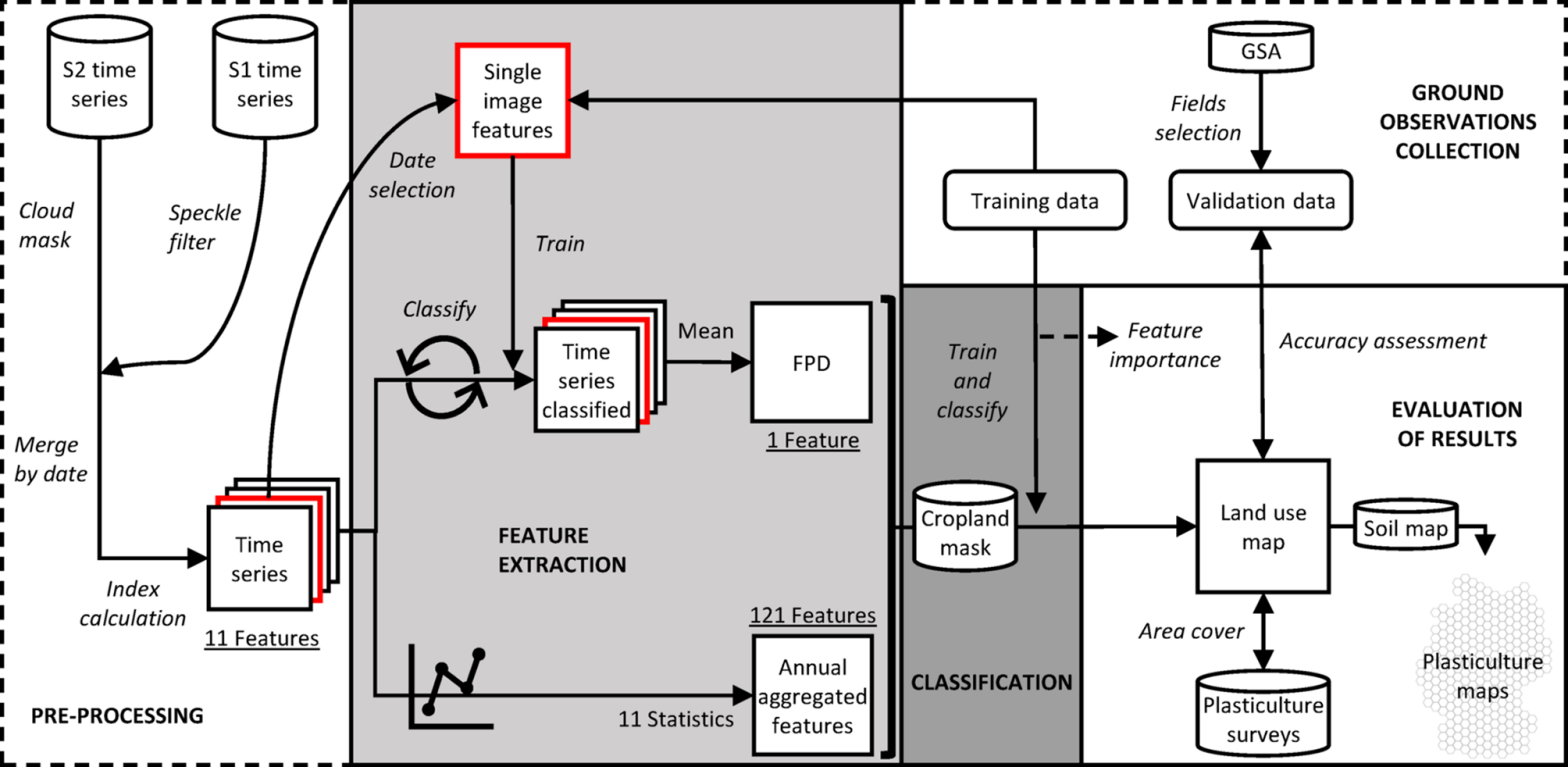
Methodological workflow, starting from the initial dataset in the upper-left corner (S2 time series and S1 time series) to the final product in the lower-right corner (plasticulture maps). Cylinder boxes represent the data used (S1 time series, S2 time series, cropland mask, GSA, soil map, plasticulture surveys). In the dashed frames, the pre-processing step (lower-left corner) and the ground observation collection step (upper-right corner). In the solid frames, the two parallel processes of feature extraction (light-grey coloured), the classification (dark-grey coloured), and the evaluation of the results (white coloured). S1: Sentinel-1; S2: Sentinel-2; GSA: geo-spatial application system; FPD: frequency of plastic detection.

The whole methodological workflow developed for the plasticulture detection algorithm is shown in Fig. 3. S1 and S2 time series available in GEE were pre-processed to obtain a fused annual time series of 11 features. The 11 pre-processed features were used as input of two parallel processes of feature extraction, yielding the frequency of plastic detection (FPD) on the one hand, and 121 annual aggregated features on the other hand. After the feature extraction, the FPD and the 121 annual aggregated features were stacked together and used as input for a random forest model^35^ to classify German arable land into PFF, PMF, and PCV. The whole workflow, except for the ground observations collection and the evaluation of the results, was developed within geemap, a Python package for the use of GEE^36^.

### Data

#### Crop reporting datasets (GSA)

Data coming from the Integrated Administration and Control System (IACS) were used in the study. IACS consists of several digital and interconnected databases, established by the EU to manage and control the payment of agricultural subsidies^37^. One of the IACS elements is the geo-spatial application system (GSA), a database containing information on agricultural practices (e.g., crop type) at the parcel level. In Germany, GSA data are managed by the Federal States. For this study, the availability of the data was limited to three Federal States in 2020 (Fig. 2), the year in which the analysis was carried on. The crop type taxonomy of the GSA database was harmonised within the three Federal States to have comparable datasets. First, all the names of the crop classes (more than 300) across the different states were homogenized. Then, some crop classes were aggregated to limit the number of classes to 54. The crop classes were aggregated based on the area covered, plant similarities, and relevance for the study (e.g., the crops belonging to the horticultural sector were kept more separated compared to cereals).

#### Cropland mask and soil map

The cropland mask used is the agricultural land layer of the digital landscape model provided by the German Federal Agency for Cartography and Geodesy^38^, related to the first half-year of 2020. The digital landscape model of the BKG divides agricultural land into nine broad sub-categories, out of which three were selected (arable land, gardening land, and fruit orchard) to mask out agricultural land uses that were not relevant for the study. After rasterization at 10 m, the obtained mask excludes 30.4% of the area covered by GSA data, mainly because of the exclusion of grassland areas (around 26%). The exclusion of GSA data was also calculated in vector format by checking the number of fields excluded, after reducing the field polygons to field centroids (Supplement 1).

The soil map of Germany 1:1,000,000 elaborated by the German Institute for Geosciences and Natural Resources^39^ was used. The map divides the territory into 71 soil mapping units and 12 soil regions, according to the German and FAO soil systems.

#### Plasticulture surveys

Based on a survey of regional experts, the Society for Plastic in Agriculture (Gesellschaft für Kunststoffe im Landbau (GKL)) estimated the area covered by agricultural plastic films in Germany during 2019^40^. The surveys documented the use of different plastic films (e.g., mulching films, fleeces, tunnel films) for seven crop types, mainly representing vegetables, strawberries, asparagus, and fruit trees. The results of the surveys are provided at the Federal State resolution up to the district resolution, but the results are missing for some regions. Bertling, et al.^41^ extrapolated the area covered by plasticulture for Germany by extending the plastic use rates calculated from the surveys through national and regional cultivation data.

#### Ground observations–training data

Google Earth (GE) very high-resolution images available in 2020 were used to manually collect and label 420 polygon samples (204 PFF, 140 PMF, 76 PCV) in two regions (Fig. 2): a first region, between Bonn and Cologne; and a second region, between Mainz and Karlsruhe. These two regions show considerable plastic use and have good availability of images in GE during 2020, ensuring training data for the plasticulture classes. Additionally, 150 polygons were randomly extracted from the GSA crop reporting dataset of North Rhine-Westphalia and equally distributed between maize, winter cereals, and winter rapeseed, which are three main crop classes that are usually not associated with plastic use in Germany. The fields were automatically labelled as PFF to increase the number and representativity of the most frequent class^42^.

#### Ground observations–validation data

**Fig. 4:**
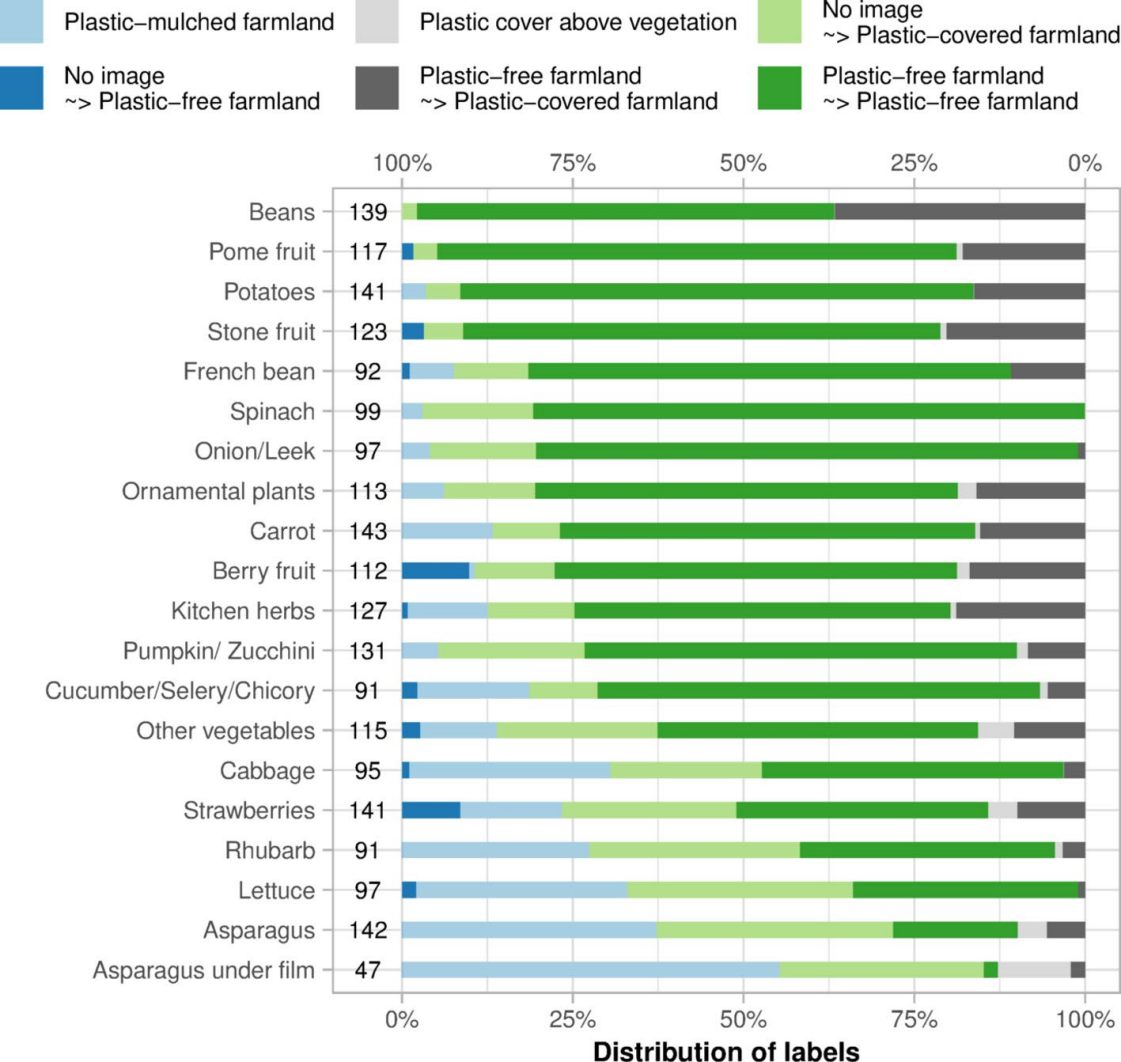
Distribution of labels in the validation data, divided by crop type. On a blue scale, fields labelled as plastic-covered with the images available in 2020. On a grey scale, fields where no image was available in 2020. On a green scale, fields labelled as plastic-free farmlands with the images available in 2020. The different greys and greens indicate whether the field was covered by plastic in the historical imagery. The number of labelled fields is indicated between the crop type names and the bars.

The validation dataset includes fields selected in the three Federal States with the GSA data (Fig. 2) and labelled through visual interpretation of GE images. The major challenge when collecting ground observations for plasticulture detection is to identify a land use that represents a rare class when compared to the whole of the agricultural land. A random selection of points on the agricultural land would lead to a strongly unbalanced dataset, while collecting validation data on local hotspots might limit the evaluation of the performances to specific field management and environmental conditions. For this reason, the GSA data were used to limit the validation data to 20 crop classes, mainly including vegetables, strawberries, and asparagus, along with other crop types in which plastic use has been documented^11,40^. For each of the three Federal States with GSA data available, 50 fields were randomly sampled within each of the 20 crop classes, ideally bringing the size of the validation data to 3000 fields (20 crop classes × 50 fields × 3 Federal States). However, when the state had less than 50 fields for a crop class, the crop class was not included in the validation data for that state to avoid under-representation. This is the case of some vegetable classes in Brandenburg and of the class ‘asparagus under film’, a crop class which refers to asparagus fields cultivated under plastic films and listed for the State of Brandenburg only, hence excluded from the States of Rhineland-Palatinate and North Rhine-Westphalia. Moreover, some fields were excluded by the applied cropland mask, finally bringing the size of the validation dataset to 2253 fields (Fig. 4). Each field was then labelled twice through visual interpretation of very high-resolution GE images. The first set of labels was assigned using the images available in 2020. If no plastic cover was found in 2020, the field was labelled as PFF. When both PMF and PCV were found in 2020, the field was labelled as PCV because of its lower presence in the study area, thus trying to ensure a more balanced dataset within the plasticulture classes.

A second set of labels was necessary to account for the low and heterogenous temporal resolution of GE images, which provide up to a few images per year depending on the region. As a result, 308 fields did not have available images in 2020 and could not be labelled. Moreover, considering that PMF, and to some extent other plastic covers, can only be seen in a very short time window, a field labelled as PFF in 2020 might have been covered with plastic on another day of the year. Therefore, the fields labelled as PFF and the fields where no image was available in 2020, were checked and labelled a second time through visual interpretation of all the other images available between 2010 and 2022, hereinafter referred to as historical imagery. During this second step, the fields were labelled according to a binary classification: plastic-covered farmland or PFF. Overall, the fields labelled as plastic-covered were more than doubled when using historical imagery (Fig. 4). In particular, the asparagus under film class, which is expected to have a plastic-covered area close to 100%, passed from 55% of PMF cover to 96% of plastic covers (Fig. 4).

#### Satellite data

The European Union’s Copernicus S1 and S2 constellations were selected as openly and freely available remote sensing data sources^43,44^. For both sensors, all the images available for Germany in 2020 between January 1st and December 31st were extracted. Overall, the selection includes 1036 acquisitions for S-1 and 2576 acquisitions from S-2, reduced to 1097 after excluding the acquisitions with a cloud cover greater than 60%. To prioritize high spatial resolution, the 10 m resolution bands (i.e., blue, green, red, near-infrared (NIR)) were selected, as previous studies have highlighted limitations with coarser resolution data^25,30,45^. With the same purpose, S1 Ground Range Detected (GRD) level-1 data were filtered to extract Interferometric Wide swath (IW) high-resolution acquisitions of VV and VH polarizations, having 10 m of pixel size. S1 data are already pre-processed by GEE to dual-polarimetric backscatter coefficients, after border and thermal noise removal, radiometric calibration, and terrain correction. When exporting outputs for the use outside of GEE, all the images were resampled to match the ETRS89-LAEA projection at 10 m resolution.

### Pre-processing

Satellite data were pre-processed to remove clouds from optical images and speckle effect from radar images. Clouds were masked out by using the cloud probability image dataset, calculated through the s2cloudless model^46,47^, while the speckle effect was reduced by applying a circular kernel filter with 50 m of smoothing radius through the ee.Image.focal_mean function in GEE. Since the optical and radar satellites have different orbits and acquisition modes, thus leading to differences in acquisition dates and geometries, each S2 pixel was stacked together with the closest S1 pixel acquired within five days, merging the observations of the two sensors into one image collection. To minimize the distance between S1 and S2 observations, both ascending and descending orbits were selected from S1.

Once the images from the two sensors were stacked together, a VV/VH ratio was calculated together with the following optical indexes:

- $$\text{NDVI }\left(\text{Normalized Difference Vegetation Index}\right)= \frac{NIR-red}{NIR+red}$$ ^48^
- $$\text{GNDVI }\left(\text{Green Normalized Difference Vegetation Index}\right)= \frac{NIR-green}{NIR+green}$$ ^49^
- $$\text{BNDVI }\left(\text{Blue Normalized Difference Vegetation Index}\right)= \frac{NIR-blue}{NIR+blue}$$ ^50^
- $$\text{MSAVI }\left(\text{Modified Soil Adjusted Vegetation Index}\right)=\frac{2NIR+1-\sqrt{{\left(2NIR+1\right)}^{2}-8\left(NIR-red\right)}}{2}$$ ^51^

Hence, at the end of the pre-processing, every image in the time series had 11 features, out of which three were derived from radar data (VV, VH, VV/VH ratio) and eight were derived from optical data (blue, green, red, NIR, NDVI, GNDVI, BDNVI, MSAVI).

### Time series analysis for different plasticulture management

**Fig. 5:**
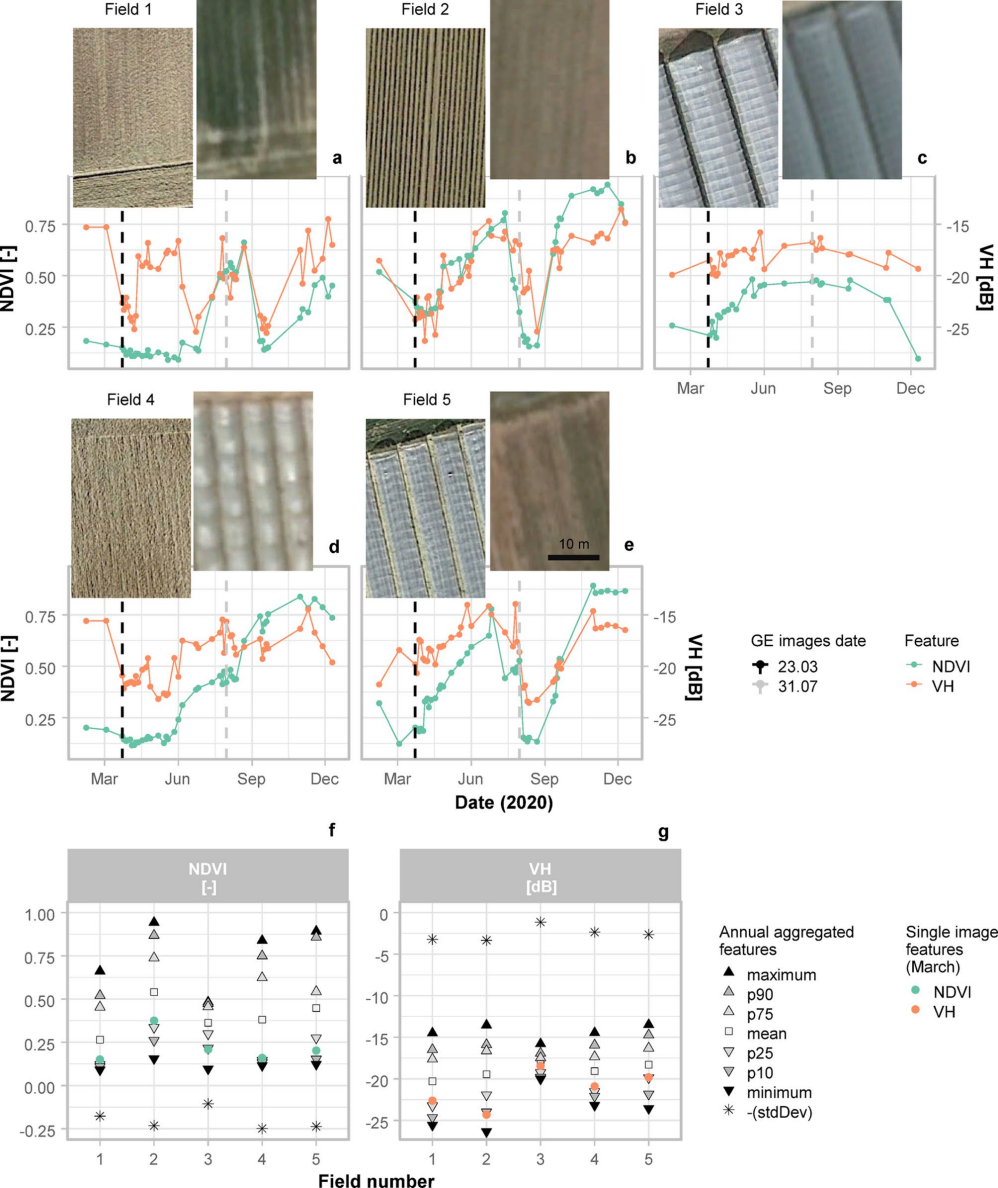
In the upper part, NDVI and VH time series for the strawberry fields represented above the plots (a-e). The images of the fields are available in Google Earth and were acquired on 23.03 and 31.07.2020, represented respectively by the black and the grey dashed lines. In the lower part, the values calculated from the annual aggregation of the features are plotted with reference to the field number and compared with the values obtained from Sentinel-1 and Sentinel-2 mosaics built with images acquired between March 23rd and March 25th (**f**–**g**). The sign of standard deviation values was reversed to enhance readability. p90: 90th percentile, p75: 75th percentile, p25: 25th percentile, p10: 10th percentile, stdDev: standard deviation, GE: Google Earth.

To introduce the feature extraction methodology and understand how the seasonal use of plastic covers affects the analysis of the time series, we extracted NDVI and VH backscatter annual time series of five strawberry fields with different plasticultural management (Fig. 5). Fields with the same crop type were selected to minimize the influence of crop-driven seasonal variability in comparing plastic uses.

When plastic covers are used throughout the whole year, the seasonal variations of NDVI and VH backscatter caused by growth cycles are hidden, and the time series are flattened (Fig. 5c). As a result, a permanent PCV can be effectively separated from a PFF (Fig. 5a) using annual aggregated features (Fig. 5f,g). Smaller and less permanent covers are more influenced by seasonal vegetation dynamics (Fig. 5d,e), especially when the plastic cover is removed. However, they show similar VH and NDVI values in single image acquisitions (Fig. 5f,g). The effect of seasonality is even more evident on a PMF covered with black films (Fig. 5b). Here, the differences arising from a comparison with PFF (Fig. 5a,f,g) appear to be mainly related to the presence of a double phenological cycle (Fig. 5b), and decoupling the seasonal variations of plastic use from environmental variables is more challenging. At the same time, PMF shows a unique combination of high NDVI and low VH backscatter in single image acquisitions (Fig. 5f,g), which can be attributed to the black-coloured films influencing the optical reflectance but not the radar backscatter. Overall, the seasonal use of plastic films is embedded in the time series together with other natural seasonal variables such as soil and vegetation conditions. While permanent plastic film use can prevail over the periodical changes of natural variables, a temporary use might be harder to detect by analysing a time series, and the analysis of single image acquisitions might be more effective. Therefore, two parallel processes of feature extraction were designed, using both annual aggregated features and single image acquisitions.

### Feature extraction

#### Annual aggregated features

The variability of feature values within the yearly time series was described by calculating the following 11 statistics at 10 m resolution: percentiles (10th, 25th, 75th, 90th), mean, maximum, minimum, interquartile range, standard deviation, the difference between 90 and 10th percentile, and the difference between maximum and minimum. Since 11 different statistical indicators were computed for each of the 11 features obtained at the end of the pre-processing, 121 annual aggregated features (11 features × 11 statistics) were extracted. In this way, we aimed at extracting features to describe annual variability and extreme values (i.e., annual aggregated features) of the land uses object of the study.

#### Frequency of plastic detection

Since plastic covers can be used for a limited time during the year, especially in the case of PMF, annual aggregated features alone might lead to training features of plasticulture classes related to a field which is a PFF for most of the year. At the same time, the ground observations in the training dataset were labelled according to one or a few very high-resolution images available during the year, and a PFF can be covered by plastic during other times of the year. Therefore, a parallel process of feature extraction has been designed to solve the above-cited training ambiguity while exploiting dense yearly time series. First, we selected cloud-free S2 images where the label assigned with GE images could be verified. The selected S2 images were acquired on March 23rd 2020 for the training region between Cologne and Bonn, and on April 4th 2020 for the training region between Mainz and Karlsruhe (Fig. 2). Then, the S2 images and the corresponding S1 acquisitions were mosaicked, and the values of the 11 features obtained at the end of the pre-processing were extracted from the polygonal areas described in paragraph 2.3.4. The extracted values were used to train a random forest with 300 decision trees and 5 variables used per split, implemented in GEE through the ‘ee.Classifier.smilRandomForest’ function. The trained classifier was used for a binary classification (0 for PFF, 1 for the plasticulture classes) of all the individual images available in the time series. As a result, the classification turned a yearly time series of satellite images into a yearly time series of classified images. The time series of classified images was finally reduced to a single feature by calculating the pixel-wise mean over the year, thus representing the frequency of plastic detection (hereinafter referred to as FPD) in the time series. The FPD has values between 0 and 1, where FPD = 0 represents a pixel that has never been classified as plastic-covered, and FPD = 1 represents a pixel that has always been classified as plastic-covered.

### Classification

After the feature extraction, the 122 features (i.e., FPD and 121 annual aggregated features) were stacked together and used as input for a second random forest classifier, trained with 300 decision trees and 10 variables used per split. The classifier was trained with the input features extracted from the polygonal areas described in paragraph 2.3.4. In a later step, the trained classifier was used to classify German arable land, selected through the cropland mask described in section "Cropland mask and soil map". After the classification, the output image was sieved by passing 4-connected pixel groups to a circle-shaped kernel with 40 m of radius, aiming at eliminating isolated pixels in the map.

### Evaluation of the results

#### Plasticulture maps and area cover

The distribution of PMF and PCV across the country was represented by calculating the area covered by the two land uses in equal hexagonal areas, where each of the hexagons covers an area of approximately 11.6 10^3^ ha, if not intersecting with the German border. Additionally, we used the FPD to highlight areas where the classification results might have been influenced by the presence of specific soil types or conditions, which resulted in a soil reflectance similar to the plastic-covered soils. We checked for the presence of hexagonal areas where the FPD correlated with the number of observations acquired in bare soil conditions. We used the following NDVI-based normalized index as a proxy estimation of the number of observations acquired in bare soil conditions:

1 $$\text{soil}\_\text{cover}= \frac{\sum_{i=1}^{n}{[(NDVI}_{i}-{NDVI}_{mid})<0]-\sum_{i=1}^{n}{[(NDVI}_{i}-{NDVI}_{mid})\ge 0]}{n}$$

where $n$ is the number of observations and ${NDVI}_{mid}$ is the central value between the maximum (${NDVI}_{max}$) and the minimum (${NDVI}_{min}$) of the NDVI:

2 $${NDVI}_{mid}= \frac{{NDVI}_{max}+{NDVI}_{min}}{2}$$

${NDVI}_{mid}$ does not aim at being an exact threshold for bare soil conditions, but it rather provides an approximate estimate of the number of observations collected in bare soil conditions. The NDVI thresholds available in the literature do not account for the presence of plastic covers, where we observed different NDVI values compared to non-covered bare soils (Fig. 5). The resulting $soil\_cover$ has values between − 1 and 1, where values greater than 0 represent pixels where the number of observations with $\text{NDVI}<{NDVI}_{mid}$ (i.e., bare soil) is bigger than the number of observations with $\text{NDVI}\ge {NDVI}_{mid}$ (i.e., vegetation). Finally, a linear regression between the FPD and $soil\_cover$ was performed and the mean R^2^ values were calculated for each hexagon. High R^2^ values mean that the frequency of plastic detection in the time series correlates with the number of observations collected in bare soil conditions. Consequently, high R^2^ values were assumed to indicate the confusion of plastic covers with uncovered bare soil, and the hexagons with high R^2^ were masked out if related to unique soil units, according to the soil map of Germany.

Finally, we used the plasticulture surveys conducted by the GKL^40^ as reference data to evaluate the differences between our results and the best currently available estimation of the plasticulture area in Germany. At the regional scale, we analysed the results in a few relevant states, while at the country scale, we compared the mapped plasticulture area with the extrapolation provided by Bertling, et al.^41^.

#### Accuracy assessment and feature importance

The classification accuracy was assessed using the centroids of the fields labelled in the validation dataset. First, the accuracy metrics (i.e., overall accuracy, producer accuracy, user accuracy) were calculated against the labels collected on the GE images available in 2020. Then, the same classification output was checked a second time against the labels collected on the historical imagery, by grouping the plasticulture classes into the plastic-covered farmland class.

The feature importance was calculated as the sum of the decrease in the Gini impurity index^52^ over all the trees in the forest, for all the 122 features used in the classification. Following the results of the feature importance, the distribution of training data values in the most important annual aggregated features is compared with the FPD and with the single image feature values used to extract it.

## Results

### Plasticulture distribution in Germany

**Fig. 6:**
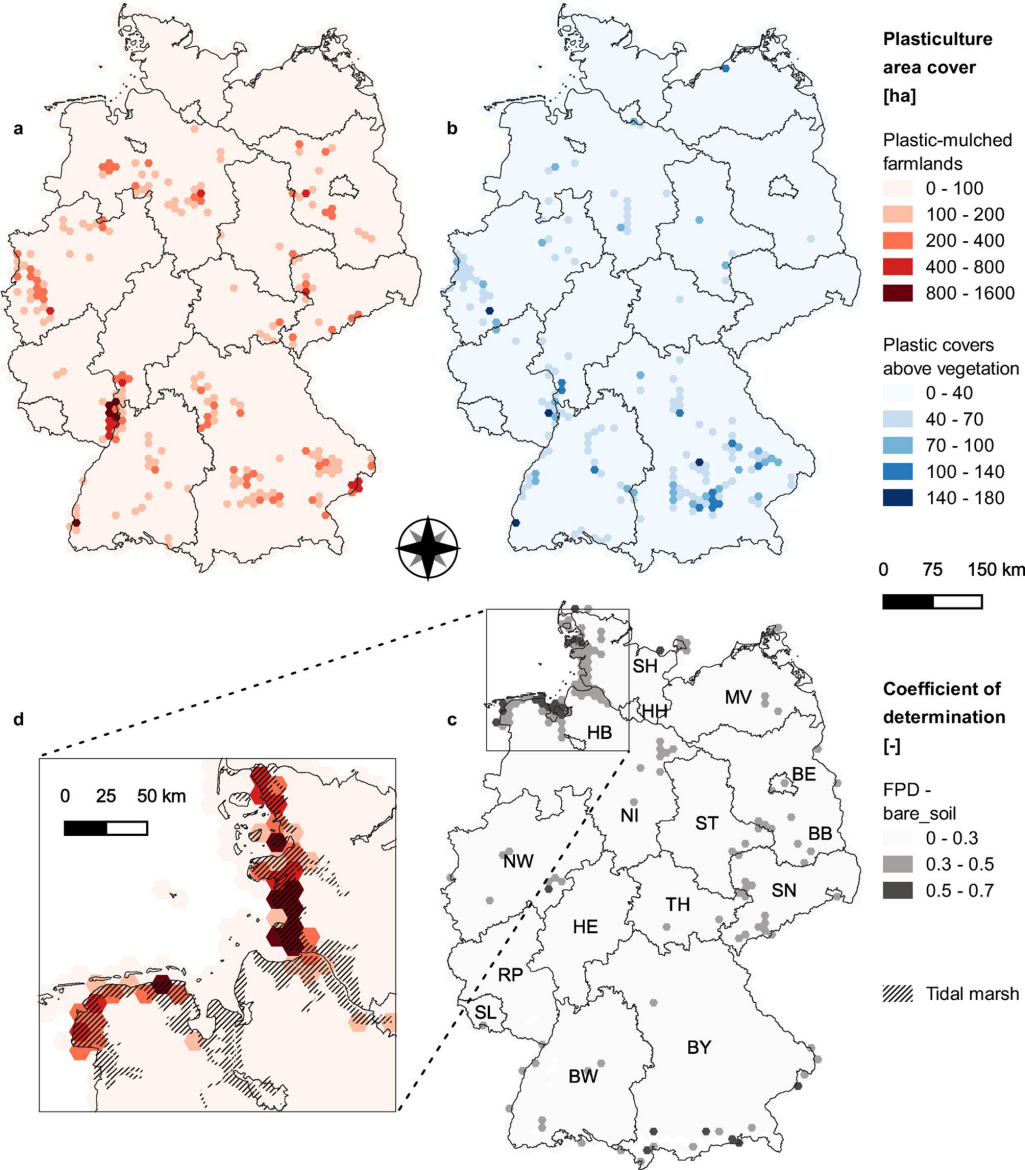
In the upper part, hexagonal maps related to the area detected [ha] as plastic-mulched farmland (**a**) and plastic cover above vegetation (**b**) in Germany, respectively represented by red and blue colour scale gradients. In the lower right corner, R^2^ values were obtained from a linear regression between FPD (frequency of plastic detection) and soil_cover (**c**). In the lower-left corner, detail of the tidal marsh area and detected plastic-mulched farmland area cover (**d**). Each hexagon has an area of approximately 11.6 10^3^ ha. BW: Baden-Württemberg, BY: Bavaria, BE: Berlin, BB: Brandenburg, HB: Bremen, HH: Hamburg, HE: Hesse, NI: Lower Saxony, MV: Mecklenburg-Vorpommern, NW: North Rhine-Westphalia, RP: Rhineland-Palatinate, SL: Saarland, SN: Saxony, ST: Saxony-Anhalt, SH: Schleswig–Holstein, TH: Thuringia. Map created using QGIS 3.12^64^.

Overall, 103.0 10^3^ ha of PMF and 37.3 10^3^ ha of PCV were detected, together representing around 1.1% of the classified area (12.9 10^6^ ha) and around 0.8% of the agricultural land (18.3 10^6^ ha) in Germany^33^. According to the classification, plasticulture is not homogenously distributed on arable land (Fig. 6a,b). Hexagons with PMF detected area greater than 200 ha represent more than one-fourth (26.5%) of the overall PMF detected, while they are distributed on 3.1% of the classified arable land. Likewise, hexagons with PCV detected area greater than 40 ha represent 28.6% of the overall PCV detected, and they are distributed on 6.6% of the classified arable land. These hotspots are mainly distributed along the Rhine Valley and in Bavaria for PMF and PCV, and in Brandenburg and Lower Saxony for PMF only (Fig. 6a,b). In general, PCV hotspots are present in regions where a high area covered by PMF was found as well, while PMF hotspots are also present in areas where PCV were not detected (Fig. 6a,b). Moreover, PCV occupy a lower area compared to PMF in hotspot regions, both in terms of absolute surface and relative surface compared to the classified arable land. Indeed, within the hexagons having more than 1000 ha of arable land, the highest PCV relative detected area is 8.0%. For the same hexagon, located around Nuremberg, in northern Bavaria, PMF cover 17.4% of the arable land. In Rhineland-Palatinate, PMF was detected on up to 33.2% of the classified arable land, corresponding to almost 1.5 10^3^ ha. Other PMF hotspots were detected in the western coastal region of Schleswig–Holstein and the northern coastal region of Lower Saxony, where the regression between the FPD and $soil\_cover$ resulted in a cluster of hexagons with moderate (0.3–0.5) to moderate-high (0.5–0.7) R^2^ values (Fig. 6c), with significance level < 0.01% (Supplement 2). In these regions, the images available in GE do not show plastic use, and the arable land is mapped with three unique soil units belonging to a tidal marsh area, according to the soil map of Germany (Fig. 6d). Consequently, the tidal marsh area was masked out in the results.

With regards to the administrative boundaries, Bavaria had the highest PCV (12.3 10^3^ ha) and PMF area detected (22.5 10^3^ ha), directly followed by Lower Saxony and North-Rhine Westphalia in terms of overall plastic use (19.8 10^3^ ha between PMF and PCV). However, while the plasticulture detection rate was moderate in Lower Saxony (1.0% of the classified area), Bavaria and North Rhine-Westphalia had plasticulture detection rates of 1.5% and 1.6% respectively. Rhineland-Palatinate had the highest area of detected plasticulture relative to the classified arable land (2.2%), just behind the City States of Berlin and Hamburg (respectively 2.6% and 3.3%). In Rhineland-Palatinate, around 27% of the vegetables were detected as PMF, while PMF detection was above 50% in asparagus and strawberry. Vegetables, strawberry, and asparagus had the highest PMF detection rate also in North Rhine-Westphalia (respectively 11.5%, 26.1%, and 61.9%), together with Rhubarb (37.5%). In Brandenburg, vegetable and strawberry cultivation areas are low, and PMF were mainly detected in asparagus and asparagus under foil classes, where PMF detection rates were 43.6% and 81.9% respectively. Overall, the PCV detection rate was lower and mainly attributed to strawberry and fruit production, where 4.2% of the area was classified as PCV.

### Comparison with plasticulture surveys

Overall, 140.3 10^3^ ha of plasticulture (PMF and PCV) were detected, while Bertling, et al.^41^ estimated a plasticulture area cover in Germany of 99.8 10^3^ ha for strawberry, asparagus, vegetables, bush berries, pome and stone fruits. Concerning plastic use in individual Federal States, the algorithm detected 34.9 10^3^ ha of plastic covers in Bavaria. According to the plasticulture surveys^40^, Bavaria is the 4th State by plasticulture area cover with 8.50 10^3^ ha of strawberry, asparagus, vegetables, bush berries, pome and stone fruits cultivated under plastic covers. However, Bavaria is divided into seven administrative districts (Lower Franconia, Middle Franconia, Upper Franconia, Upper Palatinate, Swabia, Lower Bavaria, Upper Bavaria) and the data include from two up to six of these districts, depending on the crop surveyed. In particular, plastic use data for asparagus are available for Lower and Upper Franconia only, where around 800 ha of plastic covers are estimated from the surveys, whereas asparagus was grown on a total of 4.1 10^3^ ha in Bavaria in 2016^53^. Lower Saxony is the second State by detected plasticulture area with 19.8 10^3^ ha. For the same state, the survey estimated around 12 10^3^ ha of plastic covers used in 2019, but data about plastic use in strawberries and bush berries are missing. Besides the missing data in some regions, some crop types classified in our study were not included in the surveys at all, with potatoes probably being the most relevant among them. Indeed, plastic use in potatoes has been documented to produce early potatoes^54^, and potato cultivation occupied 273.5 10^3^ ha in Germany in 2020^33^. Specifically, Bavaria and Lower Saxony are respectively the second and the first Federal States by area cultivated under potatoes (42.6 10^3^ ha and 122.2 10^3^ ha)^33^. While we do not know if plastic covers were detected in potato cultivations in Bavaria or Lower Saxony, we do know that 2.8 10^3^ ha of PMF were detected over 58.0 10^3^ ha of potato cultivations in the Federal States with GSA data available, reaching a PMF detection rate peak of 18.7% in Rhineland-Palatinate (1.4 10^3^ ha). Moreover, the use of plastic covers in potatoes was confirmed by the validation data (Fig. 4).

As for the Federal States where GSA data were available, the area of plastic covers detected was 19.8 10^3^ ha in North Rhine-Westphalia, 10.4 10^3^ ha in Rhineland-Palatinate, and 8.2 10^3^ ha in Brandenburg. In the same Federal States, the survey assessed the plasticulture area to around 15.9 10^3^ ha for North Rhine-Westphalia, 7.8 10^3^ ha for Rhineland-Palatinate, and 8.7 10^3^ ha for Brandenburg. In these three states, 8.9 10^3^ ha of plasticulture were detected in crop classes that were not included in the validation dataset, hence assumed to be plastic-free. More than half were detected in maize (4.5 10^3^ ha) and winter cereals (almost 10^3^ ha), despite their low plasticulture area cover relative to the overall area cultivated with maize and winter cereals, respectively 0.8% and 0.1%. To provide a term of comparison, the classes asparagus and asparagus under film accounted together for 6.8 10^3^ ha of detected PMF and around 90 ha of detected PCV in the same states, although having a substantially higher plasticulture detection rate. Other GSA classes not included in the validation sample with more than 1% of plasticulture detection were construction places (16.3%–147 ha), mixed crops in row cultivation (14.8%–214 ha), soybeans (3.7%–64 ha), other protein crops (2.3%–64 ha), and energy crops (1.5%–54 ha).

### Accuracy assessment

From the 1945 ground observations having at least one image available in 2020, PFF is the class with the best user accuracy and producer accuracy, and it is also the one driving the overall accuracy (85.3%) given its prevalence in the number of labelled points (Table 1). In particular, most of the fields classified as PFF were found to be correct and the resulting user accuracy is 93.7%, while the producer accuracy is 88.9%. PCV has a producer accuracy of 69.2% and it was confused with PMF in 2 observations only. PMF has a producer accuracy of 66.9% and it has been classified as PFF 88 times, while it has been classified as PCV just 4 times. The main inaccuracy is a commission error in the PMF and PCV classes, having respectively 51.8% and 65.9% user accuracy.

**Table 1:** Confusion matrixes calculated by comparing the classification results with the ground observations collected with GE images available in 2020 (upper matrix) and with GE historical imagery (lower matrix). In the lower matrix, PMF and PCV were aggregated to the plastic-covered farmland class. UA: user accuracy, PA: producer accuracy.

Ground observations 2020 images	Classification			
	Plastic free farmland	Plastic-mulched farmland	Plastic cover above vegetation	PA
Plastic free farmland	1447	171	10	88.9%
Plastic-mulched farmland	88	186	4	66.9%
Plastic cover above vegetation	10	2	27	69.2%
UA	93.7%	51.8%	65.9%	85.3%

Ground observations historical imagery	Plastic free farmland	Plastic-covered farmland		PA
Plastic free farmland	1438	98		93.6%
Plastic-covered farmland	389	328		45.7%
UA	78.7%	77.0%		78.4%

**Fig. 7:**
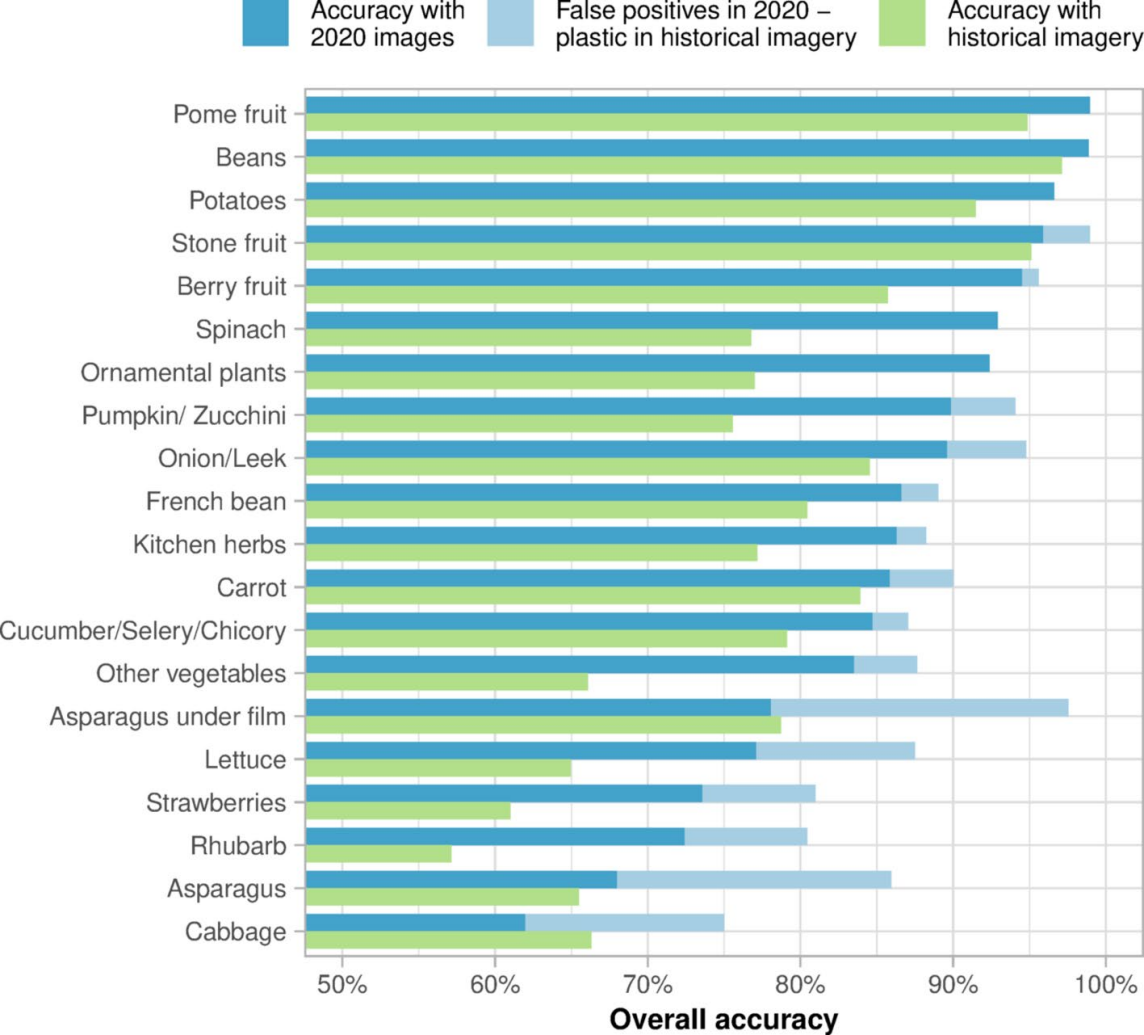
Binary classification accuracy (plastic-free farmland or plastic-covered farmland) divided by crop type for observations labelled with GE images available in 2020 (blue bars) and in historical imagery (green bars). The light blue bars represent the fields identified as false negatives in 2020 and found with plastic covers in the historical imagery.

The trend was completely reversed when comparing the results with all the 2253 observations binary labelled (i.e., PMF and PCV grouped into the plastic-covered farmland class) using the historical imagery (Table 1). While the user accuracy for the plastic-covered farmland class increased to 77.0%, the producer accuracy for the same class decreased to 45.7% and the overall accuracy decreased to 78.4%. On the one hand, 98 observations of plastic-covered farmland were labelled as PFF in 2020 but classified either as PMF (91 observations) or as PCV (8 observations), hence turned from false to true positives. Most of these observations are distributed in the asparagus (23) and asparagus under film classes (8), representing 18.0% of the observations available in 2020 for asparagus and 19.5% for asparagus under film (Fig. 7). Other classes with a relevant number of false positives found with plastic covers in historical imagery are cabbage (12), lettuce (10), and strawberry (9). On the other hand, most of the new plastic-covered observations (308) were classified as PFF and decreased the overall accuracy across almost all the crop types (Fig. 7), except for cabbage (+ 4.4%) and asparagus under film (+ 0.7%), reaching a peak of -17.4% for lettuce.

### Feature importance

**Fig. 8:**
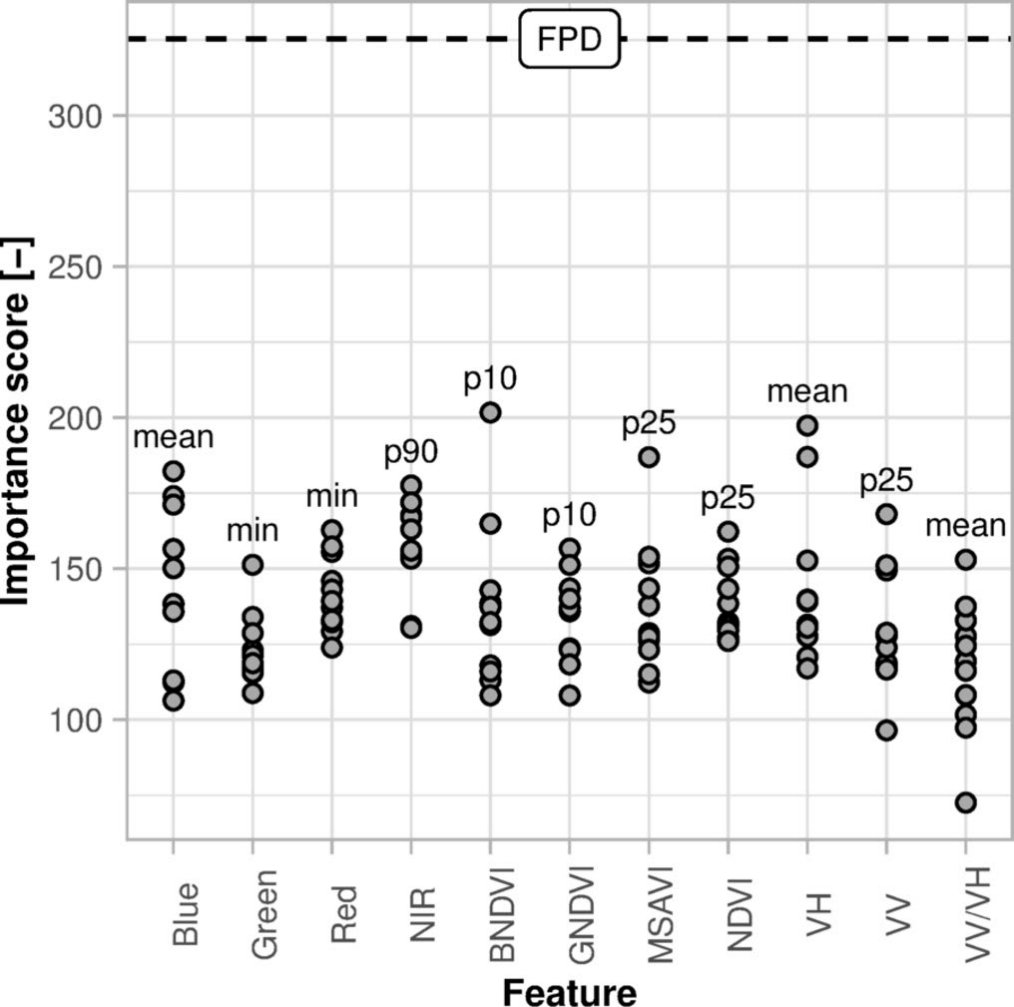
Importance score for the features used in the classification, according to the sum of the decrease in the Gini impurity index. On the x-axis, the names of the 11 bands were obtained after pre-processing. On the y-axis, feature importance score. Every point represents an annual aggregated feature. The most important feature for each band is labelled with the corresponding statistic. The importance score of the FPD (frequency of plastic detection) is represented by a dashed line. min: minimum, p90: 90th percentile, p10: 10th percentile, p25: 25th percentile.

**Fig. 9:**
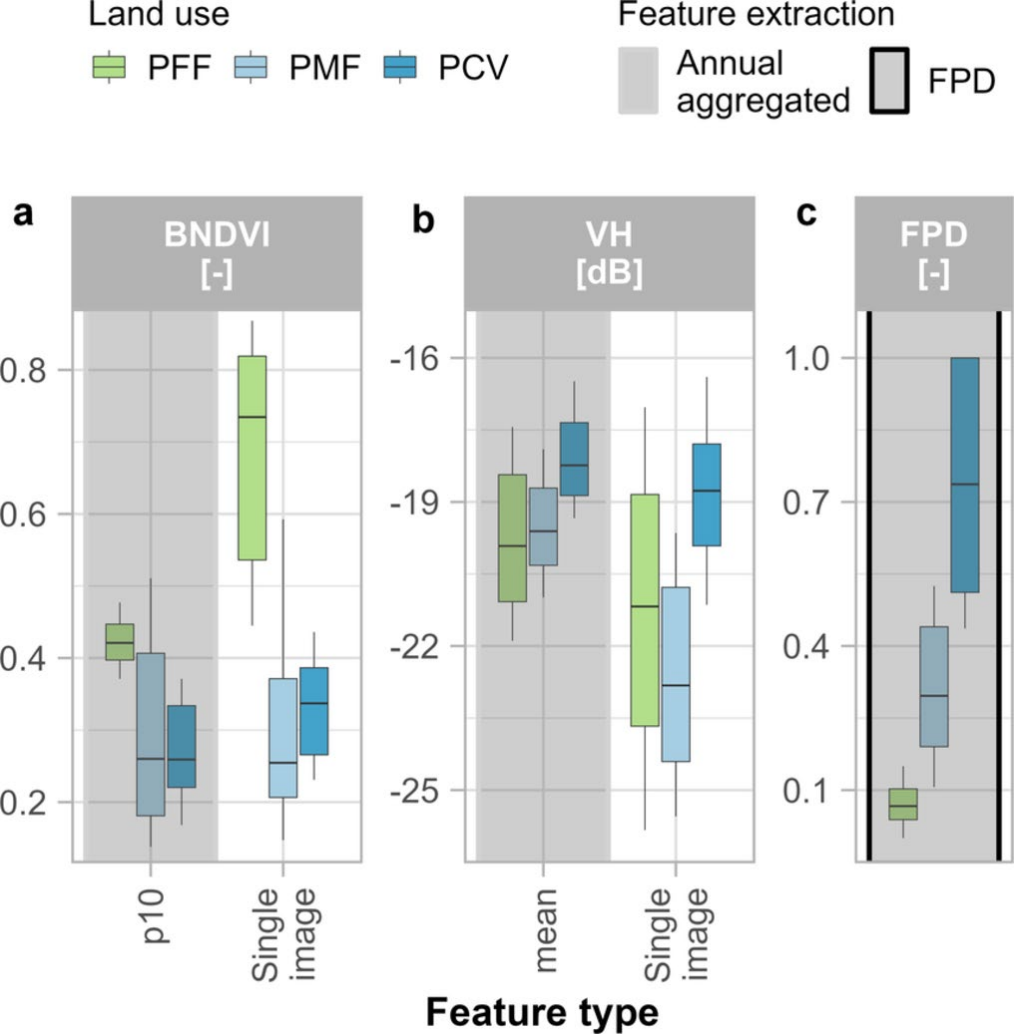
Boxplots showing the distribution of feature values for the two most important annual aggregated features (i.e., BDNVI p10, VH mean) (a-b) and the FPD (frequency of plastic detection) (**c**). For each of the annual aggregated features, the correspondent single image features used to extract the FPD (single image) are shown. The lower and upper whiskers represent respectively the 10th and 90th percentiles PFF: plastic-free farmland; PMF: plastic-mulched farmland; PCV: plastic cover above vegetation; p10: 10th percentile.

The 10th percentile of the BNDVI is the most important annual aggregated feature and the second most important feature in the overall ranking (Fig. 8). The radar features have an importance score comparable to the optical features (Fig. 8). The mean of the VH backscatter is the second most important annual aggregated feature and the most important radar feature, while VV/VH ratio shows the lower values in the feature importance score. Overall, the most important annual aggregated features have comparable importance scores, while the FPD stands out as the most important feature in the final classification (Fig. 8). Indeed, the FPD shows a higher separability of the classes compared to the most important annual aggregated features, not only between the PFF and the plasticulture classes but also within the plasticulture classes (Fig. 9). In the BNDVI, the observations used to derive the FPD (i.e., single image) guarantee a high separability between PFF and the plasticulture classes (Fig. 9a). For the same band, the best annual aggregated feature (i.e., 10th percentile) is even redundant compared to the single images (Fig. 9a). The mean values of VH backscatter generally help separate PCV from PFF (Fig. 9b). Additionally, VH single image values increase the distance between the lower quartile of the PCV class and the upper quartile of the PMF class. However, this can be attributed to the need for collecting PMF training features mostly in bare soil conditions, while PCV and PFF can be observed during any phenological stage. Hence, while the mean values are derived both from bare soils and vegetation-covered soils in all three classes, the single image values strictly refer to bare soil conditions for the PMF only.

## Discussion

### A tool for large-scale plasticulture monitoring

The results showed the main patterns and distribution of plasticulture hotspots, referring to both administrative and natural boundaries. Concerning administrative boundaries, we detected an extensive use of plastic covers in Bavaria and Lower Saxony, while an intensive use of plastic covers was observed in Rhineland-Palatinate and close to some of the biggest cities (e.g., Berlin, Hamburg, and Nuremberg). With regards to natural boundaries, we could identify the Upper and Lower Rhine Valley as the areas with the highest concentration of plastic covers. Additionally, GSA data allowed us to identify the crop types where plastic was detected in three Federal States. We confirmed vegetables, strawberries, and asparagus to be the major crop types associated with plastic use. We also identified a relevant use of plastic in potato cultivation. Despite the lower relative surface cultivated under plastic films compared to vegetables, strawberries, and asparagus, potatoes are one of the major crop types in Germany, hence plastic use should be monitored in the future. Similarly, plastic use was observed in a small fraction of maize and winter cereals, which constituted a relevant contribution to the overall plasticulture area. However, we did not include cereals in our validation data and the use of plastic covers should be confirmed by future studies.

The data available about plastic cover use in Germany (section "Plasticulture surveys") pointed at a higher plasticulture area detected by the algorithm compared to the surveys. However, it is not always clear from the surveys where multiple films are used on the same fields (e.g., mulching films and tunnel films in strawberry cultivation), thus limiting the use of the data to a retrieval of the virtual surface occupied by all the plastic films used, rather than the effective surface covered by plastic films. We analysed the differences in the most relevant states for plastic use to understand the limits of the classification and the surveys (section "Comparison with plasticulture surveys"). Specifically, the plasticulture estimates coming from the agricultural surveys are limited to strawberries, asparagus, vegetables, bush berries, pome and stone fruits, while our analysis included additional crop types, with potato probably being the most relevant crop for plastic use. Furthermore, the surveys were not conducted across the whole of Germany and some data are missing. The overall plasticulture area cover of 99.8 10^3^ ha was extrapolated by Bertling, et al.^41^ from the same surveys by accounting for the missing districts or states, but it still does not account for the missing crops. A potential overestimation of the plasticulture area in the classification could be attributed to a sparse commission error, as suggested by the larger detection of plastic covers in the Federal States with the largest classified arable land (Bavaria, Lower Saxony, and North Rhine-Westphalia). Moreover, despite the use of an agricultural land mask, small urban features such as streets, built-up areas, and construction sites were included and classified as PCV. Since the area occupied by plasticulture is still limited compared to the overall agricultural land, small misclassifications might influence country-scale dynamics.

We eliminated the influence of potential false hotspots by identifying them through a linear regression between the FPD and a proxy estimator of bare soil conditions ($soil\_cover$). We found out that the regions with moderate and moderate-high R^2^ values (Fig. 6c) corresponded to regions where plastic covers could not be observed in the available GE images. The presence of a relevant false hotspot in the northern-west coastal areas of the country was attributed to tidal marsh areas, where the soils typically have high salinity and soil organic carbon (SOC) content, probably resulting in a soil reflectance close to the plastic-covered crops. Nevertheless, high SOC is typically rare on arable land, and more than 90% of the classified area has SOC content lower than 50 g/kg, according to the European topsoil SOC map^55^. The majority of the validation data were collected in fields where SOC is lower than 50 g/kg and showed accuracy higher than 80% (Supplement 3).

We tested our classification over a large territory with different soil regions, a moderate topographic gradient, and different climates, where agricultural practices range from small-scale farming in the South to large-scale farming in the East. Altogether, the diversity of German arable land enables a wide range of applications for agricultural plastic films and values the reproducibility of our study in space and time. In this context, we proposed a methodology which greatly improves the temporal and spatial resolution of the current tools for plasticulture monitoring (e.g., surveys). To provide more accurate estimates of plasticulture area, a better understanding of the variables influencing plasticulture detection accuracy is needed (e.g., soil unit, environmental conditions, crop type). The availability of open geospatial dataset describing these variables, combined with crop declaration dataset, is an invaluable asset to increase the number and representativeness of the ground observations. As suggested by Blickensdörfer, et al.^56^, large-scale studies are not relevant just for reporting, but also for understanding the advantages and disadvantages of using satellite data in a complex and heterogeneous environment.

### Mapping a dynamic and rare land use

The reported overall accuracy of 85.3% places the methodology in the lower part of the range of accuracies of 80–95% typically observed in remote sensing detection of plastic-covered crops with medium-resolution satellite data and pixel-based approaches^21^, but it is hardly comparable with previous studies focused in spatially limited hotspot regions with lower diversity in terms of management practices and environmental conditions. For instance, thin black mulching films used in strawberry and vegetable production have never been included in a detection task before. Different film colours, frequency and time of application of the films, application on the soil surface or above the vegetation, growth rate and size of plant canopies, are all variables related to the individual crop type that could influence the accuracy of detection and that could have not been covered by the training dataset. Likewise, false plastic detection was identified in a region where ground observations were not collected. While collecting ground observations in local hotspots enables a faster collection of training data, the necessity of increasing the volume and heterogeneity of the training data might be investigated and supported by GSA data in future studies.

The algorithm showed good performances in the distinction between the plasticulture classes, where only 5.1% of the ground observations labelled as PCV were classified as PMF and 1.4% of the ground observations labelled as PMF were classified as PCV (Table 1). The presence of a relevant number of false positives in the plasticulture classes was highlighted by their low user accuracy with the ground observations labelled with GE images available in 2020. Notably, while producer accuracies of PMF and PCV are comparable, the user accuracy was particularly low for PMF. Differences in accuracy can be attributed to the higher presence of PCV in the time series. The restricted seasonal use of PMF makes them not only harder to detect in the time series (Fig. 5), but also harder to identify in the GE images used for validation. The presence of plastic covers was observed in more than half of the false positives when looking at the historical imagery. Nevertheless, comparing the classification results with the validation data binary labelled using GE historical imagery strongly increased the number of false negatives (Table 1). The oscillation between commission and omission errors in the two validation datasets highlights the challenges of collecting plasticulture ground observations, especially when dealing with a large-scale analysis and yearly time series of satellite images having a substantially higher temporal resolution compared to the reference data. However, the use of very high-resolution images is fundamental for recognising different uses of plastic covers and collecting ground observations with higher quality compared to the map product is generally a good practice for building the reference data^57^. The availability of very high-resolution images at a higher temporal resolution is key to having reliable reference data for monitoring the use of temporary plastic covers.

**Fig. 10:**
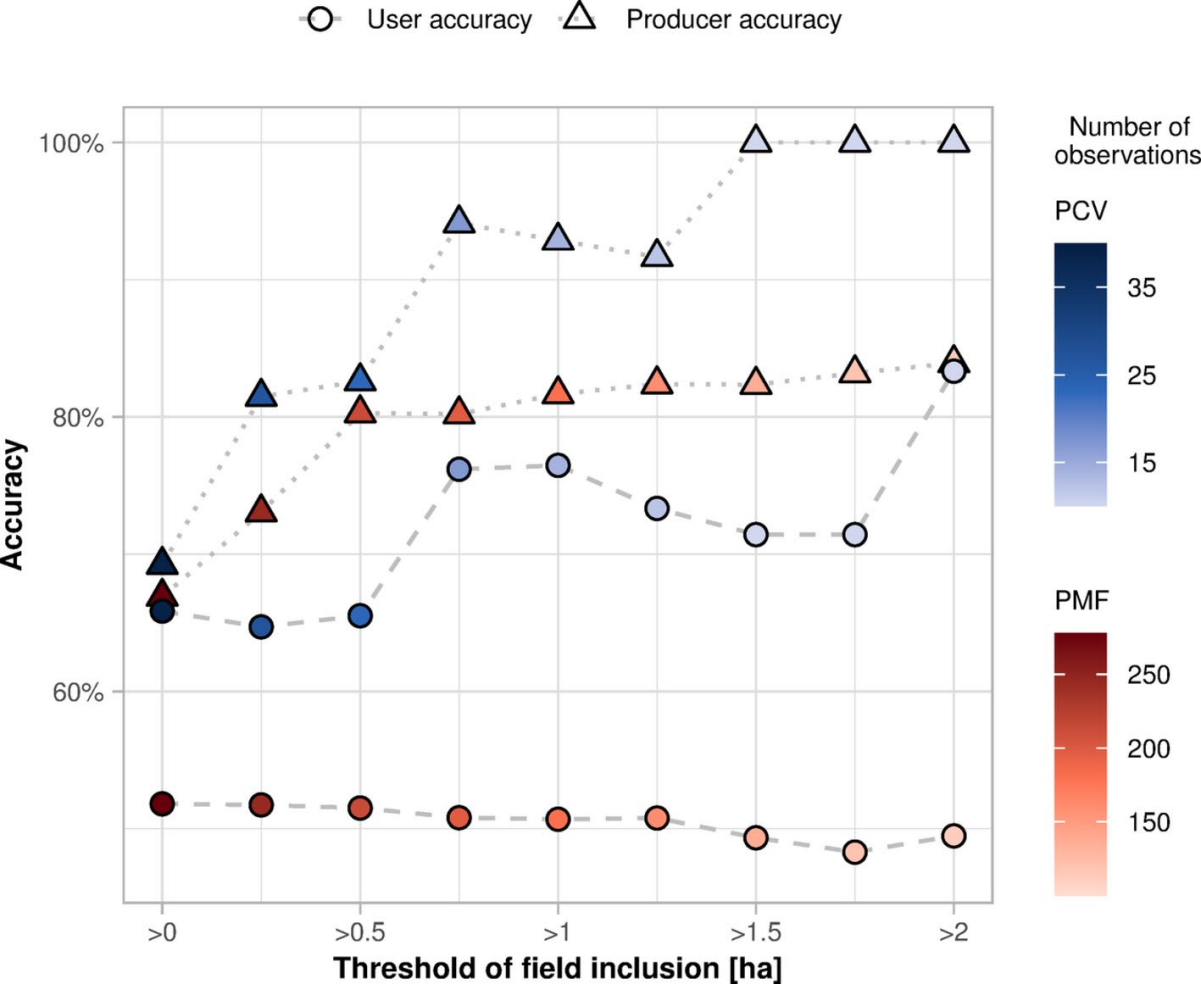
User and producer accuracy of plasticulture classes at varying thresholds of field inclusion. The accuracies were calculated at intervals of 0.25 ha. Colour gradients show changes in the number of observations used to calculate the accuracies. PCV: plastic cover above vegetation; PMF: plastic mulched farmland.

Lastly, as plasticulture was expected to be found also in small-scale farming, we did not impose a minimum field size requirement in the validation data. Even within our subset of crop types, the mean area of the fields labelled as plastic-covered is 2.7 ± 4.5 ha, compared to 3.2 ± 5.2 ha of the PFF. However, excluding fields smaller than 0.25 ha (i.e., minimum mapping unit obtained with the filter used during post-processing) increases producer accuracy of PMF and PCV, respectively, by 6.2% and 12.3% in 2020 ground observations (Fig. 10). Our ground observations indicate that fields smaller than 0.25 ha, which are potentially excluded by the post-processing, account for 14.2% of PMF and PCV observations by count but only 0.6% by area. Likewise, fields smaller than 0.5 ha represent 25.5% of PMF and PCV by count and 1.9% by area, and their exclusion increases the producer accuracy of PMF and PCV by 13.4% (Fig. 10). The accuracy further enhances for fields bigger than 1 ha (+ 14.8% for PMF and + 23.7% for PCV), but the excluded area rises to 5.1%, suggesting that a threshold of 0.5 ha provides a good trade-off between accuracy and area covered. Compared to PFF, where excluding fields smaller than 0.5 ha results in a + 2.3% gain in user accuracy but a − 2.5% loss in user accuracy, the impact of field size is more pronounced for plasticulture classes. These improvements primarily affect the producer accuracy, suggesting a higher likelihood of missing plastic-covered fields when they are smaller. Hence, while the confusion matrixes presented in the results describe the accuracy on individual parcels with homogenous field management, the evaluation of the mapped plasticulture area needs to account for decreasing omission errors with increasing field size.

### Combining annual dense time series of optical and radar data for plasticulture detection

Our methodology aimed at exploiting the high temporal and spatial resolution of the Sentinel constellation to map plasticulture. While we did not include any of the indices developed for plasticulture detection, their use might increase the accuracy of the classification, depending on the study area. The importance scores of the features used point to a combined effort of optical and radar data (Fig. 8). Since plastic films are designed to manipulate incoming solar radiation^25^, it stands to reason that plastic-covered soils can be spectrally separated from uncovered soils^58^. Nevertheless, transparent and white plastic films used for PMF may have optical properties similar to PCV, resulting in a poor distinction between the plasticulture classes^45,59^. We achieved a good distinction between the plasticulture classes, and the distinct backscatter of PCV (Figs. 5, 9), was related to the sensitivity of microwaves to the metal structures used as a support of the plastic films (Supplement 4). In our study, this resulted in high importance scores of VH especially, while the contribution of the VV/VH ratio is minor. Bigger metal structures will generally yield a higher backscatter, and more permanent metal structures will have a greater impact on the time series (Fig. 5c,d,e), making the use of radar data even more effective in regions where PCV are more diverse and frequent (e.g., Mediterranean countries).

As to PMF detection, the low dielectric constant and thickness of agricultural plastic films make them almost transparent to microwaves and hampers their use without optical data, thus explaining the poor accuracies obtained in previous studies^60,61^. However, the presence of coloured films may induce changes in optical reflection that are not followed by changes in microwave reflection, leading to linear relationships like the one described between NDVI and VH for the black PMF (Fig. 5b). Moreover, the increased microwave reflection of the plastic films in wet conditions opens possible investigations on the detectability of plastic films under dew or rain (Supplement 5–6). When the plastic films are dry, radar backscatter will be mostly influenced by the soil surface moisture and roughness. Daily and weekly fluctuations in surface moisture may be relevant in single image acquisitions and rapid mapping tasks, while the analysis of yearly time series ensures resilience to fluctuations and the acquisition of training data under both wet and dry conditions. Regarding surface roughness, it needs to be noted that both ascending and descending orbits were used for S1. Despite this did not affect our classification and no relevant differences were found between the acquisitions of different orbits (Supplement 7), most of the crops associated with plastic use are cultivated on ridges and directional scattering might happen for row orientations perpendicular to look direction^62,63^.

The FPD shows the highest separability of the classes (Fig. 9) thanks to the different frequencies of plastic cover detection in the yearly time series. Besides the highest importance score in the feature ranking (Fig. 8), this demonstrates how consistently an annual time series of images can be classified with training features coming from single acquisitions. Moreover, we produced a continuous variable that summarises the ability of a set of features to detect plastic within a time series and we used it to identify regions where the classification was influenced by the soil type.

As for annual aggregated features, substantial differences arose between the different statistical indicators used. Specifically, real values occurring in the time series (e.g., percentiles, maximum, minimum) are related, at best, to the land use object of detection, often overlaying with the training features extracted from single images. In contrast, standard deviation or mean values are more representative of the whole time series and provide information that cannot be obtained from single images, being an extremely valid training feature especially when dealing with more permanent land uses like PCV. Overall, the computational efficiency and insensitivity to overfitting of the random forest algorithm^42^ enables using a large number of input features. Nonetheless, we relied upon the computational resources provided by cloud computing, and an optimization of the input features should be sought to favour the application of the methodology to larger scales and in resource-limited contexts.

## Conclusions

The use of agricultural plastic films has been monitored at large scales through national market data or tools like agricultural surveys, occasionally providing plasticulture estimates at regional resolution. Medium-resolution satellite data are qualified to detect the use of plastic covers on individual fields, but large-scale mapping is mainly unexplored. We developed a large-scale mapping algorithm that exploits cloud computing to combine dense time series of the Sentinel-1 & -2 constellations. Our study used yearly time series to detect permanent to seasonal uses of plastic films, without limiting the detection of seasonal plastic films to specific temporal windows. This opens potential applications of the algorithm in other time frames or regions where the seasonal use of plastic films differs from our study area. Additionally, inter-annual climatic variability, changes in field management practices, or future climate change adaptation strategies might alter the temporal windows related to plastic film use. Our results also demonstrated the effectiveness of radar data as a complementary observation to optical data for plasticulture mapping, establishing the capabilities of remote sensing data combination to distinguish different types of plastic covers.

We produced an annual map of the land covered by PMF and PCV in Germany. The map greatly improves the spatial resolution of the data available on plastic use in Germany and extends the identification of plasticulture hotspots from administrative to natural boundaries. The additional use of field data like the GSA crop reporting dataset enables the monitoring of emerging trends in management practices and supports the evaluation of the results. Together with very high-resolution images, crop reporting datasets are key to analysing the uncertainties and estimating plastic film use across entire countries.

Overall, the spatial and temporal resolution of publicly and freely available satellite observations, coupled with increasing computing capabilities, qualify space-borne remote sensing as a tool for monitoring plasticulture at large scales. We propose a plasticulture mapping workflow that successfully combines geospatial and open satellite data for country-scale mapping and encourages their use for continental-scale monitoring.

## Supplementary Information

Supplementary Information.

## Data Availability

The hexagonal plasticulture map, the training data, and the Google Earth Engine scripts are available at: https://doi.org/10.5281/zenodo.14055244.

## References

[1] EIP-AGRI Focus Group. Reducing the plastic footprint of agriculture–final report. (European Commission, 2021). https://ec.europa.eu/eip/agriculture/en/focus-groups/reducing-plastic-footprint-agriculture. Accessed 29 Dec 2021.

[2] Agriculture Plastic Environment Europe. *Statistics*–*plasticulture in Europe*. https://apeeurope.eu/statistics/ (2019). Accessed 22 Dec 2023.

[3] Orzolek, M. D. in *A guide to the manufacture, performance, and potential of plastics in agriculture* (ed Michael D. Orzolek) 1–20 (Elsevier, 2017). 10.1016/b978-0-08-102170-5.00001-4

[4] Espí, E., Salmerón, A., Fontecha, A., García, Y. & Real, A. I. Plastic films for agricultural applications. *J. Plast. Film Sheet.* **22**, 85–102. 10.1177/8756087906064220 (2006).

[5] Petrovich, H. F. C. The stakes of announced growth global plasticulture and the 2018 XXI CIPA congress. *Plasticulture* **138**, 28–42 (Comité International des Plastiques en Agriculture (CIPA), 2019). https://magazine.cipa-plasticulture.com/en/produit/plasticulture-magazine-2019-n-138-en-es/. Accessed 11 Oct 2023.

[6] Lamont, W. J. in *A guide to the manufacture, performance, and potential of plastics in agriculture Plastic design library* (ed Michael D. Orzolek) 45–60 (Elsevier, 2017). 10.1016/b978-0-08-102170-5.00003-8

[7] Steinmetz, Z. et al. Are agricultural plastic covers a source of plastic debris in soil? A first screening study. *Soil* **8**, 31–47. 10.5194/soil-8-31-2022 (2022).

[8] Rehm, R., Zeyer, T., Schmidt, A. & Fiener, P. Soil erosion as transport pathway of microplastic from agriculture soils to aquatic ecosystems. *Sci. Total Environ.*10.1016/j.scitotenv.2021.148774 (2021). 34328923 10.1016/j.scitotenv.2021.148774

[9] Rezaei, M. et al. Microplastics in agricultural soils from a semi-arid region and their transport by wind erosion. *Environ. Res.*10.1016/j.envres.2022.113213 (2022). 35398314 10.1016/j.envres.2022.113213

[10] Zhang, J. et al. Effects of plastic residues and microplastics on soil ecosystems: A global meta-analysis. *J. Hazard. Mater.*10.1016/j.jhazmat.2022.129065 (2022). 35650746 10.1016/j.jhazmat.2022.129065

[11] Briassoulis, D. et al. Review, mapping and analysis of the agricultural plastic waste generation and consolidation in Europe. *Waste Manage. Res.* **31**, 1262–1278. 10.1177/0734242X13507968 (2013). 10.1177/0734242X13507968 24293230

[12] Scarascia-Mugnozza, G., Sica, C. & Russo, G. Plastic materials in European agriculture: Actual use and perspectives. *J. Agric. Eng.* **42**, 15–28. 10.4081/jae.2011.3.15 (2011).

[13] FAO. Assessment of agricultural plastics and their sustainability. A call for action. (FAO, Rome, 2021). 10.4060/cb7856en.

[14] Jiménez-Lao, R., Aguilar, F. J., Nemmaoui, A. & Aguilar, M. A. Remote sensing of agricultural greenhouses and plastic-mulched farmland: An analysis of worldwide research. *Remote Sens.*10.3390/rs12162649 (2020).

[15] Aguilar, M., Vallario, A., Aguilar, F. J., Lorca, A. G. & Parente, C. Object-based greenhouse horticultural crop identification from multi-temporal satellite imagery: A case study in Almeria. *Spain. Remote Sens.* **7**, 7378–7401. 10.3390/rs70607378 (2015).

[16] Novelli, A., Aguilar, M. A., Nemmaoui, A., Aguilar, F. J. & Tarantino, E. Performance evaluation of object based greenhouse detection from Sentinel-2 MSI and Landsat 8 OLI data: A case study from Almería (Spain). *Int. J. Appl. Earth Obs. Geoinf.* **52**, 403–411. 10.1016/j.jag.2016.07.011 (2016).

[17] Lu, L., Di, L. & Ye, Y. A decision-tree classifier for extracting transparent plastic-mulched landcover from Landsat-5 TM images. *IEEE J Sel. Top. Appl. Earth Obs. Remote Sens.* **7**, 4548–4558. 10.1109/jstars.2014.2327226 (2014).

[18] Lu, L., Tao, Y. & Di, L. Object-based plastic-mulched landcover extraction using integrated Sentinel-1 and Sentinel-2 data. *Remote Sens.*10.3390/rs10111820 (2018).

[19] Wu, C., Deng, J., Wang, K., Ma, L. & Tahmassebi, A. R. S. Object-based classification approach for greenhouse mapping using Landsat-8 imagery. *Int. J. Agric. Biol. Eng.* **9**, 79–88. 10.3965/j.ijabe.20160901.1414 (2016).

[20] Yang, D. et al. Mapping plastic greenhouse with medium spatial resolution satellite data: Development of a new spectral index. *ISPRS J. Photogramm. Remote Sens.* **128**, 47–60. 10.1016/j.isprsjprs.2017.03.002 (2017).

[21] Veettil, B. K., Van, D. D., Quang, N. X. & Hoai, P. N. Remote sensing of plastic-covered greenhouses and plastic-mulched farmlands: Current trends and future perspectives. *Land Degrad. Dev.* **34**, 591–609. 10.1002/ldr.4497 (2022).

[22] Aguilar, M. A., Jiménez-Lao, R. & Aguilar, F. J. Evaluation of object-based greenhouse mapping using WorldView-3 VNIR and SWIR data: A case study from Almería (Spain). *Remote Sens.*10.3390/rs13112133 (2021).

[23] Zhou, S. et al. A knowledge-based, validated classifier for the identification of aliphatic and aromatic plastics by WorldView-3 satellite data. *Remote Sens. Environ.*10.1016/j.rse.2021.112598 (2021).

[24] Jones, H. et al. Characterization of shortwave and longwave properties of several plastic film mulches and their impact on the surface energy balance and soil temperature. *Sol. Energy* **214**, 457–470. 10.1016/j.solener.2020.11.058 (2021).

[25] Levin, N., Lugassi, R., Ramon, U., Braun, O. & Ben-Dor, E. Remote sensing as a tool for monitoring plasticulture in agricultural landscapes. *Int. J. Remote Sens.* **28**, 183–202. 10.1080/01431160600658156 (2007).

[26] Chen, Z. et al. A convolutional neural network for large-scale greenhouse extraction from satellite images considering spatial features. *Remote Sens.*10.3390/rs14194908 (2022).

[27] Lin, J. et al. Rapid mapping of large-scale greenhouse based on integrated learning algorithm and Google Earth Engine. *Remote Sens.*10.3390/rs13071245 (2021).

[28] Tong, X. et al. Global area boom for greenhouse cultivation revealed by satellite mapping. *Nat. Food* **5**, 513–523. 10.1038/s43016-024-00985-0 (2024). 38741004 10.1038/s43016-024-00985-0

[29] Xiong, Y. et al. Large scale agricultural plastic mulch detecting and monitoring with multi-source remote sensing data: A case study in Xinjiang China. *Remote Sens.*10.3390/rs11182088 (2019).

[30] Hasituya, Chen, Z., Li, F. & Hu, Y. Mapping plastic-mulched farmland by coupling optical and synthetic aperture radar remote sensing. *Int. J. Remote Sens.* **41**, 7757–7778. 10.1080/01431161.2020.1763510 (2020).

[31] Gorelick, N. et al. Google earth engine: Planetary-scale geospatial analysis for everyone. *Remote Sens. Environ.* **202**, 18–27. 10.1016/j.rse.2017.06.031 (2017).

[32] FAO. *FAOSTAT land use*. https://www.fao.org/faostat/en/#data/RL (2020) Accessed 15 Dec 2022.

[33] Destatis. *Agricultural holdings, utilised agricultural area: Länder, years, types of land use*. https://www-genesis.destatis.de/genesis/online?operation=statistic&levelindex=0&levelid=1690882460016&code=41271#abreadcrumb (2020) Accessed 24 July 2023.

[34] Eurostat. *Glossary: Agricultural holding*. https://ec.europa.eu/eurostat/statistics-explained/index.php?title=Glossary:Agricultural_holding (Accessed 22 Dec 2023.

[35] Breiman, L. Random forests. *Mach. Learn.* **45**, 5–32. 10.1023/A:1010933404324 (2001).

[36] Wu, Q. geemap: A Python package for interactive mapping with Google Earth Engine. *J. Open Source Softw.*10.21105/joss.02305 (2020).36756303

[37] European Commission. *Integrated Administration and Control System (IACS)*. https://agriculture.ec.europa.eu/common-agricultural-policy/financing-cap/assurance-and-audit/managing-payments_en. Accessed 22 Dec 2023

[38] BKG. *Digitales Basis-Landschaftsmodell*. https://sg.geodatenzentrum.de/web_public/gdz/dokumentation/deu/basis-dlm.pdf (2020) Accessed 22 Dec 2023.

[39] BGR. *Soil map of Germany 1:1,000,000 (BÜK1000)*. https://www.bgr.bund.de/EN/Themen/Boden/Projekte/Informationsgrundlagen_abgeschlossen/BUEK1000/BUEK1000_en.html (2007) Accessed 13 July 2023.

[40] GKL. *Kunststoffe im Agrarsektor*. https://www.gkl-online.de/agrar-kunststoffe.html (2019) Accessed 3 Feb 2023.

[41] Bertling, J., Zimmermann, T. & Rödig, L. Kunststoffe in der Umwelt: Emissionen in landwirtschaftlich genutzte Böden. (Fraunhofer UMSICHT, Oberhausen, 2021). 10.24406/umsicht-n-633611.

[42] Belgiu, M. & Drăguţ, L. Random forest in remote sensing: A review of applications and future directions. *ISPRS J. Photogramm. Remote Sens.* **114**, 24–31. 10.1016/j.isprsjprs.2016.01.011 (2016).

[43] Drusch, M. et al. Sentinel-2: ESA’s optical high-resolution mission for GMES operational services. *Remote Sens. Environ.* **120**, 25–36. 10.1016/j.rse.2011.11.026 (2012).

[44] Torres, R. et al. GMES Sentinel-1 mission. *Remote Sens. Environ.* **120**, 9–24. 10.1016/j.rse.2011.05.028 (2012).

[45] la Cecilia, D., Tom, M., Stamm, C. & Odermatt, D. Pixel-based mapping of open field and protected agriculture using constrained Sentinel-2 data. *ISPRS Open J. Photogramm. Remote Sens.*10.1016/j.ophoto.2023.100033 (2023).

[46] Zupanc, A. *Improving cloud detection with machine learning*. https://medium.com/sentinel-hub/improving-cloud-detection-with-machine-learning-c09dc5d7cf13 (2017). Accessed 22 Dec 2023.

[47] Braaten, J. *Sentinel-2 Cloud Masking with s2cloudless*. https://developers.google.com/earth-engine/tutorials/community/sentinel-2-s2cloudless. Accessed 26 May 2024.

[48] Tucker, C. J. Red and photographic infrared linear combinations for monitoring vegetation. *Remote Sens. Environ.* **8**, 127–150. 10.1016/0034-4257(79)90013-0 (1979).

[49] Gitelson, A. A., Kaufman, Y. J. & Merzlyak, M. N. Use of a green channel in remote sensing of global vegetation from EOS-MODIS. *Remote Sens. Environ.* **58**, 289–298. 10.1016/S0034-4257(96)00072-7 (1996).

[50] Wang, F.-M., Huang, J.-F., Tang, Y.-L. & Wang, X.-Z. New vegetation index and its application in estimating leaf area index of rice. *Rice Sci.* **14**, 195–203. 10.1016/S1672-6308(07)60027-4 (2007).

[51] Qi, J., Chehbouni, A., Huete, A. R., Kerr, Y. H. & Sorooshian, S. A modified soil adjusted vegetation index. *Remote Sens. Environ.* **48**, 119–126. 10.1016/0034-4257(94)90134-1 (1994).

[52] Breiman, L., Friedman, J., Olshen, R. A. & Stone, C. J. *Classification and regression trees*. (Chapman & Hall/CRC, New York, 1984). 10.1201/9781315139470

[53] LfL. Erosionsschutz beim Anbau von Spargel. (2017). https://www.lfl.bayern.de/mam/cms07/publikationen/daten/informationen/erosionsschutz-anbau-spargel_lfl-information.pdf. Accessed 4 Sept 2023.

[54] Brandes, E., Henseler, M. & Kreins, P. Identifying hot-spots for microplastic contamination in agricultural soils—a spatial modelling approach for Germany. *Environ. Res. Lett.*10.1088/1748-9326/ac21e6 (2021).

[55] de Brogniez, D., Stevens, C. B. A., Jones, R. J. A., Montanarella, L. & van Wesemael, B. A map of the topsoil organic carbon content of Europe generated by a generalized additive model. *Eur. J. Soil Sci.* **66**, 121–134. 10.1111/ejss.12193 (2015).

[56] Blickensdörfer, L. et al. Mapping of crop types and crop sequences with combined time series of Sentinel-1, Sentinel-2 and Landsat 8 data for Germany. *Remote Sens. Environ.*10.1016/j.rse.2021.112831 (2022).

[57] Olofsson, P. et al. Good practices for estimating area and assessing accuracy of land change. *Remote Sens. Environ.* **148**, 42–57. 10.1016/j.rse.2014.02.015 (2014).

[58] Ibrahim, E. & Gobin, A. Sentinel-2 recognition of uncovered and plastic covered agricultural soil. *Remote Sens.*10.3390/rs13214195 (2021).

[59] Feng, Q. et al. Mapping of plastic greenhouses and mulching films from very high resolution remote sensing imagery based on a dilated and non-local convolutional neural network. *Int. J. Appl. Earth Obs. Geoinf.*10.1016/j.jag.2021.102441 (2021).

[60] Hasituya, Chen, Z., Li, F. & Hongmei,. Mapping plastic-mulched farmland with C-band full polarization SAR remote sensing data. *Remote Sens.*10.3390/rs9121264 (2017).

[61] Liu, C.-A., Chen, Z., Wang, D. & Li, D. Assessment of the X- and C-band polarimetric SAR data for plastic-mulched farmland classification. *Remote Sens.*10.3390/rs11060660 (2019).

[62] Wegmüller, U. et al. Progress in the understanding of narrow directional microwave scattering of agricultural fields. *Remote Sens. Environ.* **115**, 2423–2433. 10.1016/j.rse.2011.04.026 (2011).

[63] Blanchard, B. J. & Chang, A. T. C. Estimation of soil moisture from Seasat SAR data. *J. Am. Water Resour. As.* **19**, 803–810. 10.1111/j.1752-1688.1983.tb02803.x (1983).

[64] QGIS.org. QGIS Geographic Information System. QGIS Association (2020) http://www.qgis.org.

