# Supplementary material for: Plasticulture detection at the country scale by combining multispectral and SAR satellite data
Source: Sci Rep. 2025 Apr 2;15:11339. doi: 10.1038/s41598-025-93658-2 (PMC11965282; doi:10.1038/s41598-025-93658-2)
Supplement: Supplementary file 1 — Supplementary Information. [file 41598_2025_93658_MOESM1_ESM.docx]

Supplement 1 – Evaluation of the LPIS data excluded by the use of the BKG cropland mask. Analysis carried out on area (columns 2 to 13) and number of fields excluded (columns 14 and 15).

|  | Rasterization and intersection of the two datasets | | | | | | | | | | | | LPIS fields conversion to centroids | |
| --- | --- | --- | --- | --- | --- | --- | --- | --- | --- | --- | --- | --- | --- | --- |
|  |  | LPIS area covered by DLM sub-categories | | | | | | | | | Area excluded | | Fields excluded | |
| Crop type | LPIS area [ha] | Arable land | Orchard | Hop | Grassland | Orchard meadow | Gardening land | Tree nursery | Vineyard | Fruit orchard | Excluded from DLM | Excluded from mask | LPIS | Excluded from LPIS - mask intersection |
| Ackergras | 79196 | 80% | 0% | 0% | 18% | 0% | 0% | 0% | 0% | 0% | 1% | 20% | 36524 | 28% |
| Alfalfa grass | 30425 | 90% | 0% | 0% | 7% | 0% | 0% | 0% | 0% | 0% | 3% | 10% | 6558 | 18% |
| Asparagus | 7564 | 96% | 0% | 0% | 1% | 0% | 0% | 1% | 0% | 1% | 0% | 3% | 3109 | 5% |
| Asparagus under foil | 3081 | 98% | 0% | 0% | 1% | 0% | 0% | 0% | 0% | 1% | 1% | 1% | 325 | 2% |
| Beans | 14436 | 97% | 0% | 0% | 2% | 0% | 0% | 0% | 0% | 0% | 0% | 3% | 3756 | 5% |
| Berry fruit | 1810 | 42% | 0% | 0% | 3% | 0% | 2% | 3% | 1% | 46% | 4% | 11% | 1327 | 25% |
| Cabbage | 4071 | 97% | 0% | 0% | 1% | 0% | 0% | 0% | 0% | 1% | 1% | 2% | 1629 | 6% |
| Carrot | 7681 | 99% | 0% | 0% | 1% | 0% | 0% | 0% | 0% | 0% | 0% | 1% | 1853 | 3% |
| Christmas trees | 1680 | 29% | 0% | 0% | 5% | 0% | 0% | 54% | 0% | 1% | 11% | 70% | 1487 | 74% |
| Clover grass | 30352 | 83% | 0% | 0% | 15% | 0% | 0% | 0% | 0% | 0% | 1% | 16% | 11720 | 25% |
| Construction places | 902 | 90% | 0% | 0% | 8% | 0% | 0% | 0% | 0% | 0% | 2% | 10% | 583 | 20% |
| Cucumber/Selery/Chicory | 1997 | 99% | 0% | 0% | 0% | 0% | 0% | 0% | 0% | 0% | 1% | 1% | 616 | 7% |
| Energy crops | 3658 | 88% | 0% | 0% | 10% | 0% | 0% | 0% | 0% | 0% | 2% | 12% | 1618 | 23% |
| French bean | 1264 | 99% | 0% | 0% | 0% | 0% | 0% | 0% | 0% | 0% | 0% | 1% | 382 | 5% |
| Greening / landscape elements | 30349 | 86% | 0% | 0% | 9% | 0% | 0% | 0% | 0% | 0% | 4% | 14% | 101169 | 23% |
| Kitchen herbs | 1554 | 96% | 0% | 0% | 2% | 0% | 0% | 0% | 0% | 0% | 1% | 4% | 674 | 12% |
| Lettuce | 1826 | 98% | 0% | 0% | 1% | 0% | 1% | 0% | 0% | 0% | 0% | 1% | 654 | 7% |
| Linseed (flax, linseed) | 1111 | 98% | 0% | 0% | 2% | 0% | 0% | 0% | 0% | 0% | 0% | 2% | 184 | 6% |
| Lupins | 8834 | 97% | 0% | 0% | 2% | 0% | 0% | 0% | 0% | 0% | 0% | 3% | 1243 | 8% |
| Maize | 582202 | 96% | 0% | 0% | 3% | 0% | 0% | 0% | 0% | 0% | 0% | 4% | 140254 | 7% |
| Mixed crops in row cultivation | 1446 | 87% | 0% | 0% | 3% | 0% | 4% | 1% | 0% | 1% | 4% | 8% | 1326 | 28% |
| Mixed crops with seed mixture | 1668 | 87% | 0% | 0% | 7% | 0% | 0% | 1% | 0% | 0% | 3% | 12% | 1182 | 25% |
| Mixture legumes / cereals | 7481 | 93% | 0% | 0% | 7% | 0% | 0% | 0% | 0% | 0% | 0% | 7% | 2339 | 11% |
| Mustard | 1842 | 85% | 0% | 0% | 13% | 0% | 0% | 0% | 1% | 0% | 1% | 15% | 439 | 31% |
| No use | 69388 | 71% | 0% | 0% | 23% | 0% | 0% | 0% | 1% | 1% | 4% | 28% | 62871 | 34% |
| No value | 3905 | 20% | 0% | 0% | 15% | 0% | 0% | 0% | 0% | 0% | 65% | 80% | 55082 | 43% |
| Onion/Leek | 5806 | 99% | 0% | 0% | 0% | 0% | 0% | 0% | 0% | 0% | 0% | 1% | 1848 | 2% |
| Ornamental plants | 1106 | 85% | 0% | 0% | 3% | 0% | 7% | 1% | 1% | 1% | 2% | 7% | 952 | 21% |
| Other arable fodders | 1755 | 92% | 0% | 0% | 7% | 0% | 0% | 0% | 0% | 0% | 1% | 7% | 1002 | 14% |
| Other areas | 33163 | 39% | 0% | 0% | 15% | 0% | 0% | 0% | 2% | 0% | 44% | 61% | 57249 | 72% |
| Other cereals | 5426 | 54% | 0% | 0% | 25% | 0% | 0% | 0% | 1% | 0% | 19% | 46% | 5741 | 75% |
| Other crops | 1069 | 92% | 0% | 0% | 6% | 0% | 0% | 0% | 0% | 1% | 1% | 7% | 546 | 17% |
| Other oilseeds | 1133 | 95% | 0% | 0% | 3% | 0% | 0% | 0% | 1% | 0% | 1% | 5% | 299 | 24% |
| Other permanent crops | 61196 | 3% | 0% | 0% | 1% | 0% | 0% | 0% | 94% | 1% | 1% | 96% | 208536 | 98% |
| Other protein crops | 3170 | 83% | 0% | 0% | 16% | 0% | 0% | 0% | 0% | 0% | 1% | 17% | 2163 | 23% |
| Other vegetables | 4454 | 96% | 0% | 0% | 1% | 0% | 0% | 0% | 0% | 1% | 1% | 3% | 1909 | 19% |
| Peas | 19885 | 98% | 0% | 0% | 2% | 0% | 0% | 0% | 0% | 0% | 0% | 2% | 4373 | 5% |
| Permanent grassland | 970234 | 10% | 0% | 0% | 85% | 0% | 0% | 0% | 0% | 0% | 4% | 90% | 593094 | 86% |
| Pome fruit | 4570 | 11% | 0% | 0% | 2% | 1% | 0% | 5% | 1% | 78% | 2% | 11% | 3978 | 21% |
| Potato | 57966 | 99% | 0% | 0% | 1% | 0% | 0% | 0% | 0% | 0% | 0% | 1% | 16427 | 6% |
| Pumpkin/ Zucchini | 2373 | 95% | 0% | 0% | 3% | 0% | 0% | 0% | 0% | 1% | 1% | 4% | 1417 | 13% |
| Rhubarb | 982 | 95% | 0% | 0% | 1% | 0% | 1% | 0% | 0% | 1% | 1% | 3% | 870 | 9% |
| Soybeans | 1735 | 98% | 0% | 0% | 1% | 0% | 0% | 0% | 0% | 0% | 1% | 2% | 437 | 4% |
| Spinach | 1543 | 99% | 0% | 0% | 0% | 0% | 0% | 0% | 0% | 0% | 0% | 1% | 423 | 1% |
| Stone fruit | 3585 | 14% | 0% | 0% | 2% | 0% | 0% | 2% | 1% | 80% | 1% | 6% | 879 | 23% |
| Strawberry | 4286 | 94% | 0% | 0% | 1% | 0% | 1% | 0% | 0% | 2% | 1% | 3% | 2189 | 5% |
| Sugar beet | 74516 | 99% | 0% | 0% | 0% | 0% | 0% | 0% | 0% | 0% | 0% | 1% | 20109 | 1% |
| Summer cereals | 101093 | 94% | 0% | 0% | 5% | 0% | 0% | 0% | 0% | 0% | 0% | 6% | 42057 | 11% |
| Sunflowers | 11663 | 98% | 0% | 0% | 2% | 0% | 0% | 0% | 0% | 0% | 0% | 2% | 1598 | 13% |
| Topinambur | 138 | 85% | 0% | 0% | 12% | 0% | 0% | 0% | 0% | 0% | 3% | 15% | 152 | 32% |
| Tree nurseries (without soft fruit) | 3968 | 17% | 0% | 0% | 1% | 0% | 2% | 75% | 0% | 2% | 2% | 79% | 2194 | 71% |
| Trees | 17326 | 4% | 0% | 0% | 4% | 0% | 0% | 0% | 0% | 0% | 91% | 96% | 40385 | 89% |
| Winter cereals | 1113180 | 98% | 0% | 0% | 2% | 0% | 0% | 0% | 0% | 0% | 0% | 2% | 257682 | 5% |
| Winter oilseed rape | 156848 | 98% | 0% | 0% | 2% | 0% | 0% | 0% | 0% | 0% | 0% | 2% | 30944 | 5% |


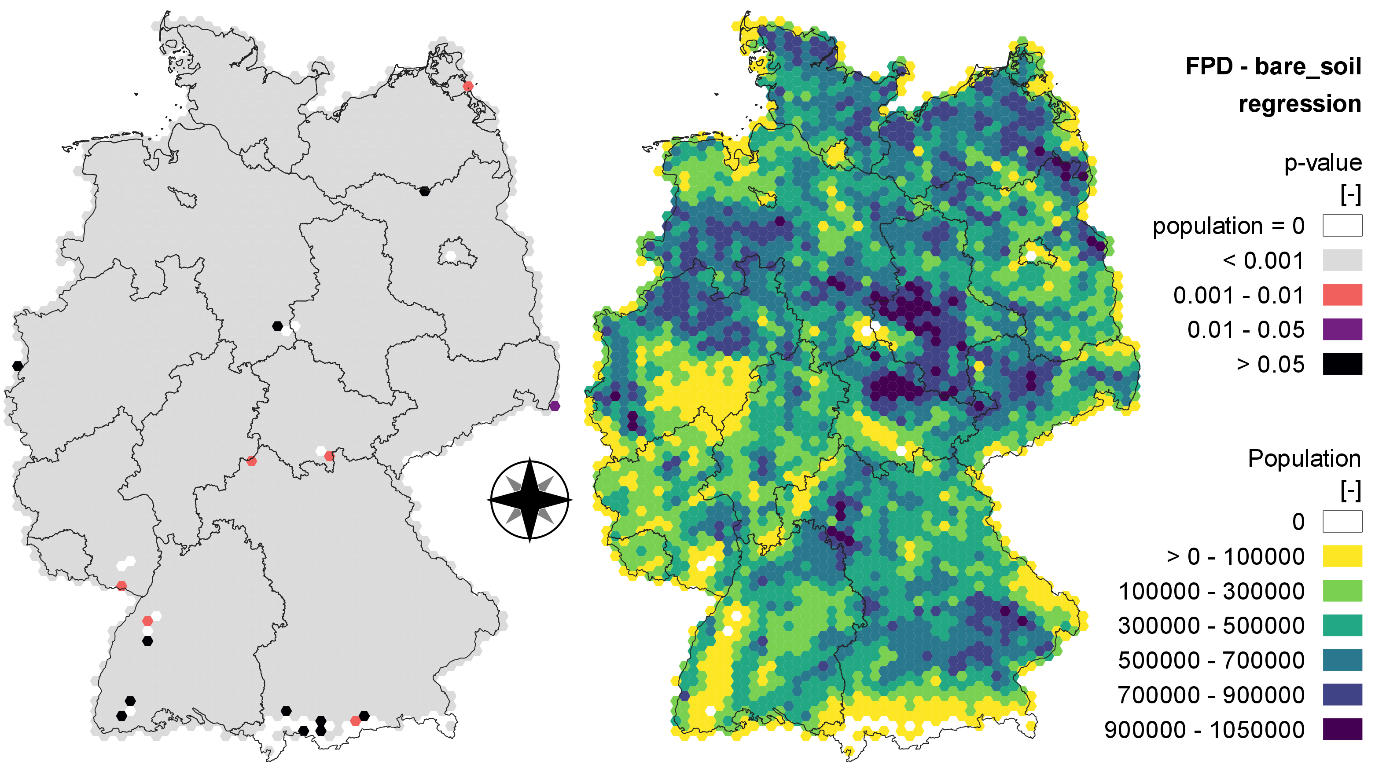


Supplement 2- On the left, p-value of the linear regression between FPD and soil_cover. Highly significant hexagons are represented in light grey, non-significant hexagons in any other color. On the right, population (number of pixels) of the regression between FPD and soil_cover. Each hexagon has an area of approximately 11.6 10^3^ ha. Map created using QGIS 3.12.[^1^](#_ENREF_1)


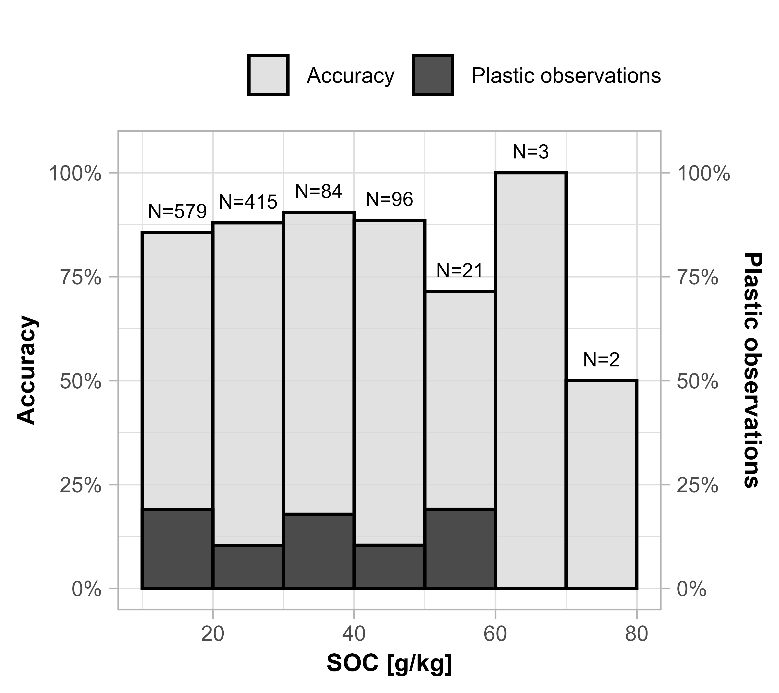


Supplement 3 - Overall accuracy of detection for different SOC (soil organic carbon) intervals. The accuracy was calculated for equal width bins of 10 g/kg on the ground observations collected with GE images available in 2020, where the field size is greater than 0.5 ha. On top of the bars is the number (N) of observations available for the SOC interval.


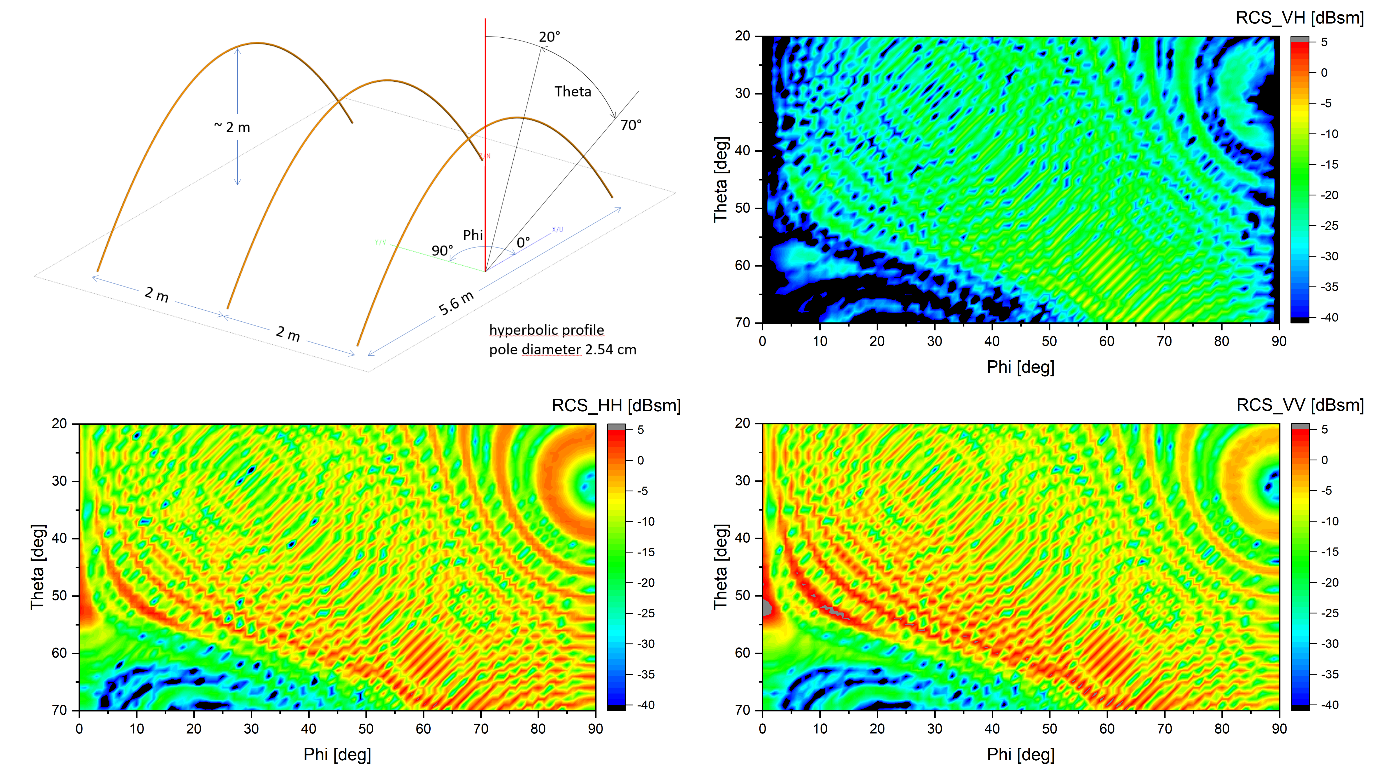


Supplement 4 – RCS characteristics of PCV structures for Sentinel-1 observation scenario. On the upper-left corner, the structure of the metal poles used to simulate the Sentinel-1 backscattering values of a typical metal frame standing below agricultural plastic foils in strawberry tunnels. Starting from the upper-right corner clockwise, VH, VV and HH backscatter coefficient values variation for different incidence angles (theta) and orientation of the tunnel (phi).


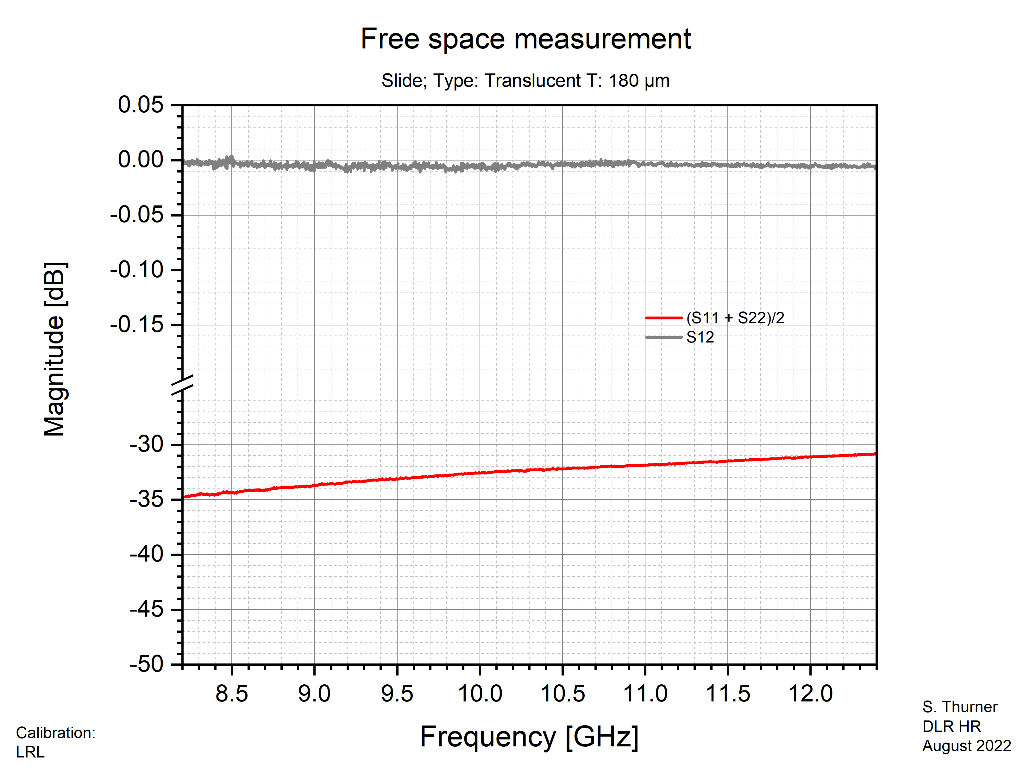


Supplement 5 - Microwave reflection (S11) and transmission (S12) of agricultural plastic film in dry conditions. A 180 µm thick LDPE film was used for the experiment.


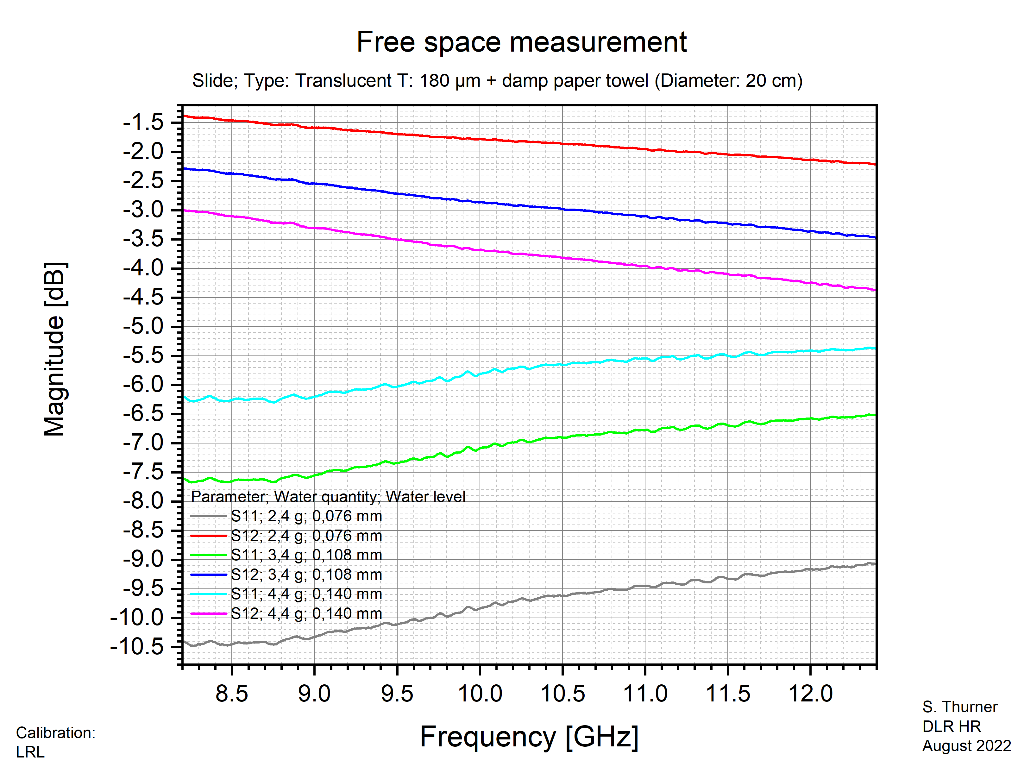


Supplement 6 - Microwave reflection (S11) and transmission (S12) of agricultural plastic film in wet conditions. A 180 µm thick LDPE film was used for the experiment. A wet paper towel was placed behind the film to simulate the presence of water.


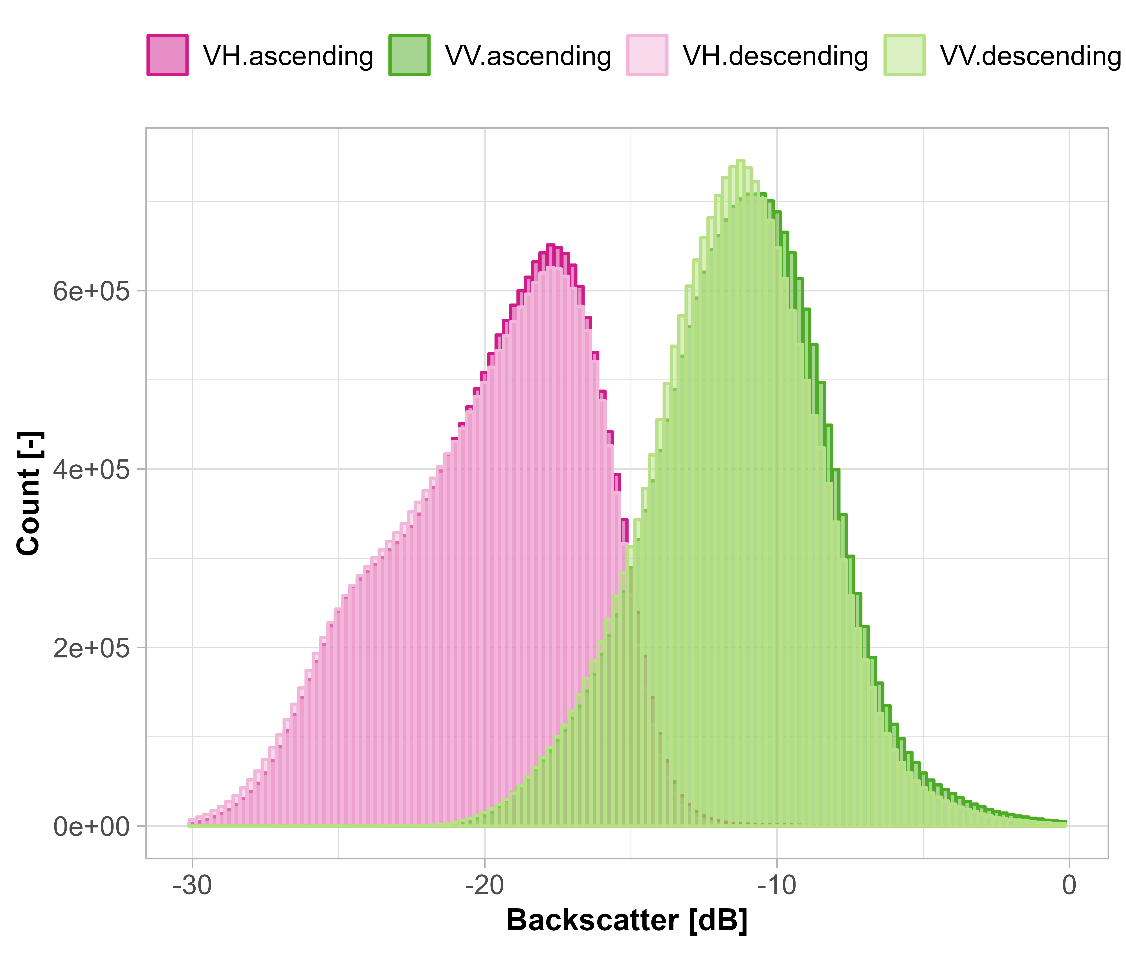


Supplement 7 - Histograms of Sentinel-1 VV and VH backscatter coefficients for ascending and descending orbits, related to the following crop types: strawberry, asparagus, potato. The pixel values were extracted from the polygons included in the validation data, using the Sentinel-1 images available between January 1^st^ 2020 and December 31^st^ 2020.

**References**

1 QGIS.org. QGIS Geographic Information System. QGIS Association (2020) <http://www.qgis.org>.
